# Birthweight, Type 2 Diabetes Mellitus, and Cardiovascular Disease: Addressing the Barker Hypothesis With Mendelian Randomization

**DOI:** 10.1161/CIRCGEN.117.002054

**Published:** 2018-06-06

**Authors:** Daniela Zanetti, Emmi Tikkanen, Stefan Gustafsson, James R. Priest, Stephen Burgess, Erik Ingelsson

**Affiliations:** 1 Division of Cardiovascular Medicine, Department of Medicine (D.Z., E.T., E.I.),; 2 Division of Cardiology, Department of Pediatrics (J.R.P.); 3 and Stanford Cardiovascular Institute (D.Z., E.I.); 4 Stanford University School of Medicine, CA. Department of Medical Sciences, Molecular Epidemiology and Science for Life Laboratory, Uppsala University, Sweden (S.G., E.I.).; 5 MRC Biostatistics Unit and Department of Public Health and Primary Care, University of Cambridge, United Kingdom (S.B.).

**Keywords:** cardiovascular disease, diabetes mellitus, type 2, genetics, hypertension, obesity

## Abstract

**Background:**

Low birthweight has been associated with a higher risk of hypertension, type 2 diabetes mellitus (T2D), and cardiovascular disease. The Barker hypothesis posits that intrauterine growth restriction resulting in lower birthweight is causal for these diseases, but causality is difficult to infer from observational studies.

**Methods:**

We performed regression analyses to assess associations of birthweight with cardiovascular disease and T2D in 237 631 individuals from the UK Biobank. Further, we assessed the causal relationship of such associations using Mendelian randomization.

**Results:**

In the observational analyses, birthweight showed inverse associations with systolic and diastolic blood pressure (β, −0.83 and −0.26; per raw unit in outcomes and SD change in birthweight; 95% confidence interval [CI], −0.90 to −0.75 and −0.31 to −0.22, respectively), T2D (odds ratio, 0.83; 95% CI, 0.79–0.87), lipid-lowering treatment (odds ratio, 0.84; 95% CI, 0.81–0.86), and coronary artery disease (hazard ratio, 0.85; 95% CI, 0.78–0.94), whereas the associations with adult body mass index and body fat (β, 0.04 and 0.02; per SD change in outcomes and birthweight; 95% CI, 0.03–0.04 and 0.01–0.02, respectively) were positive. The Mendelian randomization analyses indicated inverse causal associations of birthweight with low-density lipoprotein cholesterol, 2-hour glucose, coronary artery disease, and T2D and positive causal association with body mass index but no associations with blood pressure.

**Conclusions:**

Our study indicates that lower birthweight, used as a proxy for intrauterine growth retardation, is causally related with increased susceptibility to coronary artery disease and T2D. This causal relationship is not mediated by adult obesity or hypertension.

## Abstract 2

Supplemental Digital Content is available in the text.

**See Editorial by Laina and Stellos**

**CLINICAL PERSPECTIVE**

Low birthweight has been associated with a higher risk of hypertension, type 2 diabetes mellitus, and cardiovascular disease in epidemiological studies. The Barker hypothesis posits that intrauterine growth restriction resulting in lower birthweight is causal for these diseases, but causality and mechanisms are difficult to infer from observational studies. We address this important question with Mendelian randomization analysis to shed light on biological mechanisms behind these complex traits. In traditional observational analyses, self-reported birth weight was inversely associated with blood pressure, coronary artery disease, and type 2 diabetes mellitus and directly associated with body mass index and body fat percentage. Using Mendelian randomization, we established that lower birthweight was causally related to higher low-density lipoprotein cholesterol and 2-hour glucose and higher risk of coronary artery disease and type 2 diabetes mellitus. Further, our study suggests that increased birth weight is causally associated with increased body mass index but not causally associated with blood pressure. This is the largest Mendelian randomization study of birthweight to date, and it indicates that intrauterine growth restriction, as reflected by lower birthweight, is causally and directly related to increased susceptibility to coronary artery disease and type 2 diabetes mellitus in adulthood. This causal relationship is not mediated by adult obesity or hypertension. Our study supports the notion that population-level interventions improving prenatal nutrition and growth may improve cardiometabolic disease profiles later in life.

The association between low birthweight and increased risk of coronary artery disease (CAD) in adult life was first demonstrated by the British epidemiologist David Barker in a landmark paper in the *Lancet* in 1989.^1^ This observation was later extended using a longitudinal cohort study of 8760 participants with growth trajectories during childhood.^2^ In this study, individuals with a low birthweight increased their weight rapidly after 2 years of age and had increased risk of insulin resistance and CAD in adult life. In 1992, Barker proposed that these relationships could be explained by what he called the thrifty phenotype hypothesis^3^ attributing the association between poor fetal and infant growth and subsequent increased cardiovascular risk to arise from a compensatory response to nutritional deprivation in early life, resulting in permanent changes in glucose-insulin metabolism and somatic growth lasting into adulthood. Decreased insulin secretion and increased insulin resistance in combination with effects of obesity, aging, and physical inactivity are the most important factors leading to type 2 diabetes mellitus (T2D),^3^ but they are also independent risk factors for CAD, stroke, and hypertension.^4^

Still, it is not yet clear whether birthweight plays a causal role in the development of these outcomes as posited in the Barker hypothesis or whether other phenomena, such as confounding factors (maternal smoking, socioeconomics level, ethnicity), have resulted in spurious associations in previous observational studies. We wanted to investigate causal mechanisms using the Mendelian randomization (MR) approach. This method has the ability to infer a causal relationship between a risk factor and a disease, using genetic markers as a proxy for a modifiable exposure. In the case of birthweight, it can be considered as a summary measure reflecting several intrauterine exposures that collectively influence fetal growth. In this MR study, we used birthweight-associated variants as a proxy for intrauterine growth to examine whether reduced intrauterine growth contributes causally to later life complex diseases. Two smaller prior MR studies indicated a causal association between low birthweight and T2D^5^ but not with lipids or CAD.^6^ However, these studies were hampered by weak instrumental variables including only 5 and 7 single-nucleotide polymorphisms (SNPs), respectively, resulting in limited statistical power. Furthermore, these studies did not address the relationship of birthweight with other important cardiovascular diseases and risk factors, including atrial fibrillation (AF), ischemic stroke (IS), blood pressure, body mass index (BMI), waist-to-hip ratio (WHR), high-density lipoproteins (HDL), low-density lipoprotein (LDL), triglycerides, 2-hour glucose, fasting glucose, and fasting insulin.

The aims of the present study were to (1) describe the relationships of self-reported birthweight to several cardiovascular traits in 237 631 participants of the UK Biobank (UKB) and (2) delineate any causal relationships between birthweight and CAD, AF, IS, and T2D, and risk factors for these diseases (systolic blood pressure [SBP] and diastolic blood pressure [DBP], BMI, WHR, HDL, LDL, triglycerides, 2-hour glucose, fasting glucose, and fasting insulin) by 2-sample MR analysis using summary statistics from the largest available genome-wide association study (GWAS) meta-analyses.

## Methods

The authors declare that all data are publicly available in the UKB repository.^7^ The UKB study was approved by the North West Multi-Centre Research Ethics Committee, and all participants provided written informed consent. Data on birthweight; CAD; AF; IS; SBP and DBP; BMI and WHR; HDL, LDL, and triglycerides; T2D; 2-hour glucose, fasting glucose, and fasting insulin have been contributed by EGG (Early Growth Genetics),^8^ CARDIoGRAMplusC4D (Coronary ARtery DIsease Genome wide Replication and Meta-analysis [CARDIoGRAM] plus The Coronary Artery Disease [C4D] Genetics),^9^ AFGen (Atrial Fibrillation Genetics),^10^ ISGC (International Stroke Genetics Consortium),^11^ ICBP (International Consortium for Blood Pressure),^12^ GIANT (Genetic Investigation of Anthropometric Traits),^13,14^ GLGC (Global Lipids Genetic Consortium),^15^ DIAGRAM (Diabetes Genetics Replication and Meta-Analysis),^16^ and MAGIC (Meta-Analysis of Glucose and Insulin Related Traits Consortium)^17^ investigators, respectively.

### Study Sample

The UKB is a longitudinal cohort study of >500 000 individuals aged 40 to 69 years initiated in the United Kingdom in 2006–2010.^7^ We included 237 631 participants who knew their birthweight; to focus on the linear effects of birthweight, we limited analysis to individuals reporting birthweight to be within 2.5 and 4.5 kg and excluded individuals with cardiovascular disease prior enrollment (Methods section and Table I in the Data Supplement). We used UKB for our observational analyses, as well as to perform a GWAS of SBP and DBP (as publically available summary statistics were adjusted for BMI) to create an instrumental variable (IV) for the MR analyses. Cardiovascular outcomes for observational studies were defined using the *International Classification of Diseases* codes (details in Methods section in the Data Supplement). The exposure of interest was self-reported birthweight.

For our MR analyses, we used publicly available GWAS summary statistic of birthweight ^8^ as exposure and of CAD,^9^ AF,^10^ IS,^11^ SBP and DBP (adjusted for BMI),^12^ BMI,^13^ WHR,^14^ HDL, LDL, triglycerides,^15^ T2D,^16^ 2-hour glucose,^17^ fasting glucose, and fasting insulin^18^ as outcomes. Details on the GWAS consortia, number of samples, proportion of variance explained, and statistical power for MR analysis are presented in the Table.

**Table.**
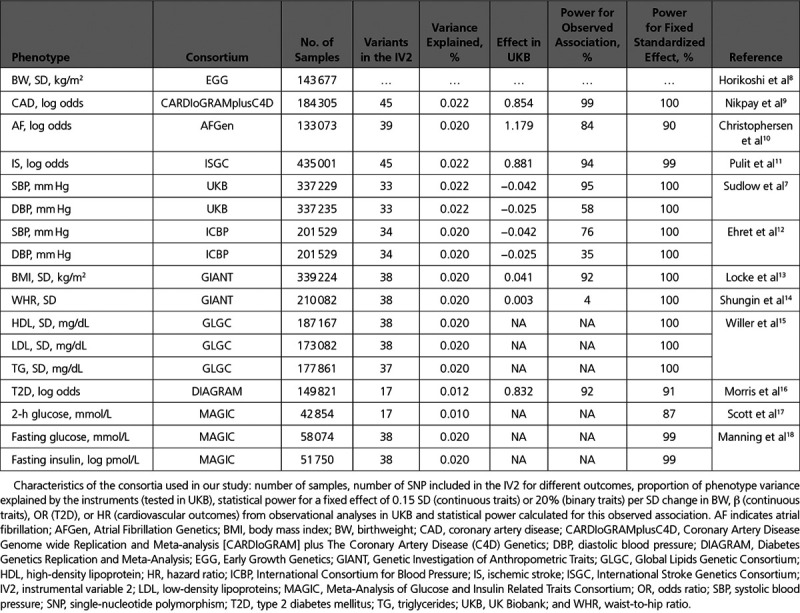
Description of Data Used and Statistical Power for Mendelian Randomization Analyses

### Statistical Methods

#### Observational Analyses

After confirming normal distribution of all continuous variables, we performed multivariable linear regression models to assess associations of birthweight with SBP, DBP, BMI, body fat, and WHR and multivariable logistic regression models to study associations of birthweight with T2D and lipid medications. Multivariable-adjusted Cox proportional hazards models were performed to assess associations of birthweight with CAD, AF, IS, hemorrhagic stroke, and heart failure events, separately during a median follow-up of 6.1 years (maximum 6.7 years). We use the DAGitty web tool (http://dagitty.net/dags.html) to systematically construct our multivariable model adjusting for confounders (Figure I in the Data Supplement). All association analyses were adjusted for age, sex, region of the UKB assessment center, ethnicity, maternal smoking, and Townsend index. We assessed evidence of nonlinear effects of birthweight on different outcomes using spline regression models. We excluded any violation of the proportionality assumption in our Cox regression analyses (all *P*>0.30) by Schoenfeld residuals test. All observational analyses were performed in the UKB.

#### Mendelian Randomization

We performed 2-sample MR analyses using publically available consortia data, except for blood pressure where we performed a GWAS in UKB. We assessed the causal relationships of birthweight with CAD, AF, IS, and T2D and risk factors for these diseases (SBP, DBP, BMI, WHR, HDL, LDL, triglycerides, 2-hour glucose, fasting glucose, and fasting insulin) using the 2-sample MR approach.^19,20^ To minimize the risk of pleiotropy affecting our results, we performed analyses using 3 different IVs:

IV1: Including up to 58 independent lead variants (excluding the insulin-like growth factor 2 [IGF2] locus because of imprinting; see Methods section in the Data Supplement) from the GWAS of birthweight performed by the EGG consortium^8^;
IV2: Including up to 46 variants after exclusion of 12 variants associated with CAD, AF, IS, and T2D at GWAS significance; any confounders at GWAS significance; or with any of the confounders or CAD, AF, IS, and T2D at a *P* value lower than the *P* value for association with birthweight (Figure II and Table II in the Data Supplement). These associations were estimated in UKB.
IV3: Excluded 1 to 9 heterogeneous variants (different for each outcome; Figure III in the Data Supplement). We performed a stepwise downward model selection in which SNPs were iteratively removed from the risk score until the heterogeneity test was no longer significant at the prespecified threshold (*P*<0.05) using the R package gtx.

We decided a priori that IV2 would constitute our main model (balancing high statistical power and low risk of pleiotropy) but included IV1 to maximize power and IV3 to decrease risk of pleiotropy in sensitivity analyses.

We performed 2-sample MR using 4 separate methods to estimate causal effects for binary and continuous outcomes: the standard inverse-variance weighted regression, the robust penalized inverse-variance weighted, and 2 robust regression methods, the weighted median-based method and Egger regression.^20^ We performed leave-one-out sensitivity analyses to identify whether a single SNP was driving an association. To further address whether birthweight had a causal effect on CAD and T2D independently of BMI, we used a multivariate MR weighted regression-based method, in which the causal effects of multiple related risk factors can be estimated simultaneously.^21,22^

We estimated statistical power for the different MR analyses (Table) using sample sizes and variance explained specific for each analysis and an α threshold of 0.05 for 2 different effect sizes: (1) assuming a fixed effect across phenotypes of 0.15 SD (continuous outcomes) or 20% (odds ratio, 1.2; dichotomous outcomes) and (2) for traits that were available in UKB, the effect size from observational analyses.

MR analyses were conducted with the R packages TwoSampleMR^23^ and MendelianRandomization.^24^ Power for MR analyses was estimated with an online tool by Burgess (https://sb452.shinyapps.io/power/). Observational analyses were conducted with the R package Survival (version 3.3.0).

A flow chart of the different data sources used in this study is shown in Figure IV in the Data Supplement. A detailed description of material and methods can be found in the Methods section in the Data Supplement.

## Results

In UKB, the mean age at baseline was 55.0 years (SD, 8.1 years) and 61% of subjects were females. During follow-up, 5542 incident cardiovascular disease cases occurred in participants free from the disease at baseline (2656 CAD; 1580 AF; 688 IS; 363 hemorrhagic stroke; and 255 heart failure events; Tables I and III in the Data Supplement).

### Observational Analyses

The results from observational analyses are summarized in Figure (full results in Table III in the Data Supplement). We observed strong inverse associations between birthweight and blood pressure, CAD, T2D, and lipid-lowering treatment. In contrast, we observed strong and positive associations between birthweight and BMI and body fat percentage. After adjusting for multiple testing (12 traits), the associations were nonsignificant for WHR, AF, IS, hemorrhagic stroke, and heart failure. We excluded nonlinear associations between birthweight and any outcomes tested (*P*>0.05) by spline regression (Figure V in the Data Supplement).

**Figure.**
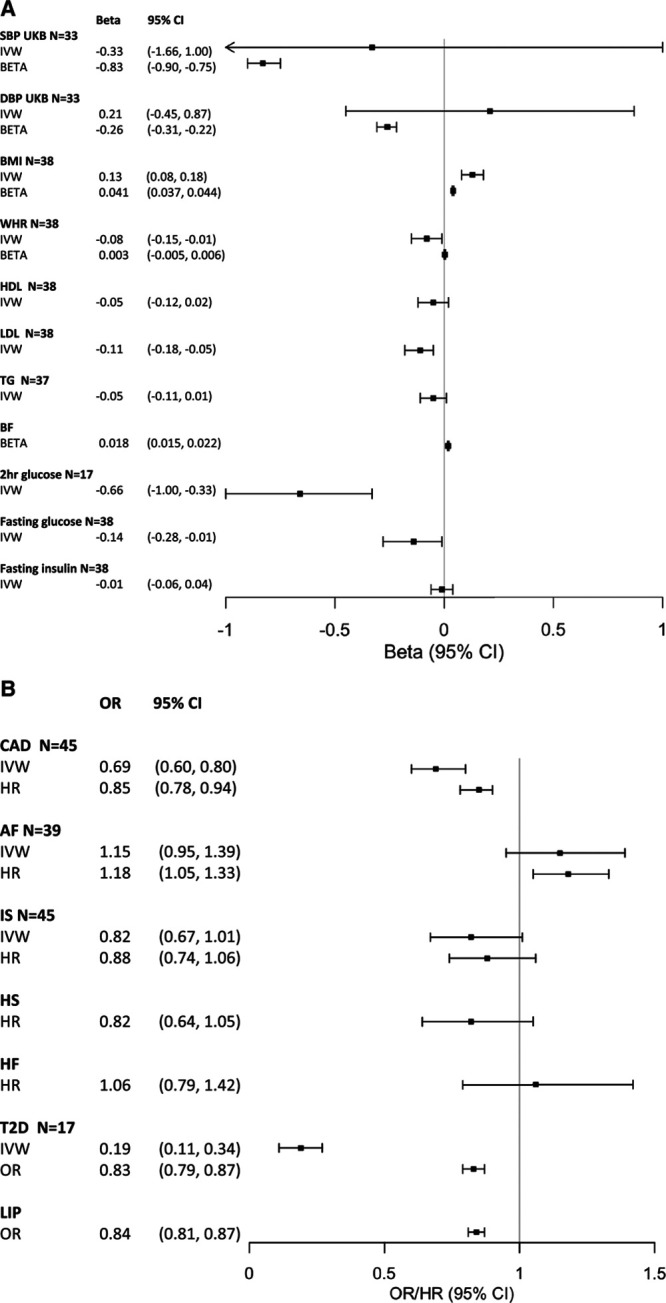
Inverse-variance weighted (IVW) estimates from Mendelian randomization (MR) analyses and association results (BETA/ hazard ratio [HR]/odds ratio [OR]) from observational analyses of birth weight (BW) with cardiovascular outcomes in UK Biobank (UKB) using multivariable-adjusted linear and logistic regression, and multivariable-adjusted Cox proportional hazards models. **A**, Continuous outcomes: systolic blood pressure (SBP) and diastolic blood pressure (DBP) in UKB, body mass index (BMI), waist-to-hip ratio (WHR), high-density lipoprotein (HDL), low-density lipoprotein (LDL), triglycerides (TG), body fat percentage (BF), 2-h glucose, fasting glucose, and fasting insulin. **B**, Binary outcomes: coronary artery disease (CAD), atrial fibrillation (AF), ischemic stroke (IS), hemorrhagic stroke (HS), heart failure (HF), type 2 diabetes mellitus (T2D), and lipid medications (LIP). The β values from linear regression represent SD change in outcome variable per SD change in BW, except for SBP and DBP where they represent the outcome in raw unit (mm Hg) per SD change in BW. MR analyses were based on the 46 variants included in the instrument variable 2 using data sources listed in the Table. All effects for the IVW (β or OR) are given in original units as provided by the consortia. Model adjustment: age, sex, region of the UKB assessment center, ethnicity, maternal smoking, and Townsend index. CI indicates confidence interval; and N, number of variants included in the instrument variable.

### Mendelian Randomization

In our main analyses (inverse-variance weighted using the 46-SNP IV [IV2]), we found evidence of causal associations of birthweight with BMI, LDL, 2-hour glucose, CAD, and T2D (Figure). The direction of the effect was negative for all the above outcomes (ie, higher birthweight was associated with lower risk and vice versa), with the exception of BMI, where higher birthweight was associated with higher BMI. We did not find evidence of causal effect of birthweight on HDL, triglycerides, fasting insulin, AF, and IS.

The leave-one-out sensitivity analysis did not highlight any heterogeneous SNPs with a large effect on the results. After excluding heterogeneous SNPs in the IV3, our analysis showed no significant heterogeneity and no significant directional horizontal pleiotropy (all *P*>0.05; Figure VI in the Data Supplement).

The analyses using penalized robust inverse-variance weighted, MR Egger, and weighted median methods consistently yielded similar effect estimates but as expected with wider confidence intervals, especially for Egger regression (Table IV and Figure VII in the Data Supplement). Further, sensitivity analyses using alternative IVs with higher power (IV1) and lower risk of pleiotropy (IV3) also provided similar results (Table IV in the Data Supplement).

The mediation analysis using the multivariate MR weighted regression-based method showed an independent association between birthweight and CAD, as well as between birthweight and T2D, not mediated by BMI in either case. The direction of the effect detected was consistent with our main MR analyses (Table IV in the Data Supplement).

We had good statistical power to detect causal associations for all traits when assuming a fixed effect across phenotypes of 0.15 SD (continuous outcomes) or 20% (odds ratio, 1.2; dichotomous outcomes). When using the effect sizes from observational analyses of traits that were available in UKB, the power was adequate for all traits except DBP and WHR.

## Discussion

### Principal Findings

In this study of 237 631 individuals from the general population, we used self-reported birthweight as a proxy for fetal development to analyze downstream consequences of intrauterine growth restriction. We describe the association of birthweight with incidence of T2D and 5 cardiovascular outcomes (CAD, AF, IS, hemorrhagic stroke, and heart failure) and cardiometabolic risk factors (blood pressure, BMI, body fat, and WHR), and we identify a causal role of birthweight in the development of several cardiometabolic diseases. Our principal findings are several. First, in our observational study, we established that self-reported birthweight displays strong inverse associations with blood pressure, CAD, and T2D and strong direct associations with BMI and body fat. Second, our MR analyses indicate that low birthweight, used as a proxy for intrauterine growth retardation, is causally related to higher risk of LDL and 2-hour glucose and higher CAD and T2D in adults. This highlights the influence of prenatal determinants of fetal growth on the development of cardiometabolic diseases in adulthood. Third, our study suggests high birthweight to be causally associated with increased BMI but not causally associated with blood pressure. Taken together and considering the different direction of the causality for BMI and CAD/T2D (higher birthweight increases BMI; lower birthweight increases CAD and T2D), our results suggest a plausible causal association of intrauterine growth restriction and low birthweight with risk for CAD and T2D, an association that does not seem to be mediated by obesity or hypertension.

In their initial description of the thrifty phenotype hypothesis,^3^ Barker and Hales proposed that BMI would be a possible mediator of the associations detected between low birthweight and adult T2D and CAD. The hypothesized primary effect of BMI was supported by evidence from both population and experimental studies linking low birthweight with predisposition to an increased risk of metabolic diseases, such as T2D,^25–29^ hypertension,^30,31^ and CAD.^32^ However, in our study and in prior observational analyses, higher birthweight is associated with obesity (a universally recognized correlate of cardiometabolic disease) in both childhood^33,34^ and adulthood.^8,35^ Our findings suggest a plausible causal association of low birthweight with CAD and T2D, which is uniquely independent of the relationship between high birthweight and increased BMI. Consistent with our observed effects of low birthweight on risk for CAD and T2D independent of adult obesity, a recent study of black women failed to detect a causal role for BMI in mediating the increased risk for T2D in adult life among individuals with low birthweight.^36^ New models for how risk for cardiometabolic disease in adulthood is directly conferred by growth restriction in utero without a compensatory change in BMI are needed to explain our observation of a direct causal relationship.

Explicit in the Barker hypothesis and explored by the experimental literature^37,38^ is a model in which prenatal growth stress leads to metabolic reprogramming beginning in utero. In the setting of prenatal malnutrition, the fetus is hypothesized to shift toward insulin resistance to allow for maximum uptake of available energy and nutrients. In this hypothesis, the persistence of insulin resistance after parturition might then trigger rapid postnatal growth with the concomitant potential for increased long-term risk of T2D, obesity, and CAD in adulthood.^25,39^ However, our findings support a separate direct causal link between intrauterine growth restriction and long-term risk for cardiometabolic disease, which does not involve adult obesity. Consistent with our detection of a causal relationship, one prior report using IV analyses, but with much fewer variants, also described a direct causal association between low birthweight and T2D.^5^

In contrast to our results, Au Yeung et al,^6^ reported no causal association between birthweight and CAD. However, this study was based on a weak IV consisting of 7 SNPs, explaining only 0.45% of the variance in birthweight (in contrast to our score that explained 2.2% of the variance), resulting in limited statistical power of 56% suggested by post hoc calculations. In this context, it is also worth mentioning the genetic correlation analyses of birthweight with several health-related traits, published in the recent GWAS for birthweight used to create IVs for our MR study.^8^ As in our study, they reported strong positive genetic correlations with BMI, and inverse genetic correlations with CAD and T2D. In contrast to our MR results, they highlight a negative genetic correlation with SBP. This discrepancy is probably related to the different methods used. Indeed, they used the linkage-disequilibrium score regression model,^40^ which use all GWAS summary statistics of the traits of interest to estimate the genetic correlations, while MR methods are based on a much smaller number of variants, aiming to decrease the risk of horizontal pleiotropy driving associations.

### Clinical Implications

Our observation that low birthweight is causally related to LDL, 2-hour glucose, CAD, and T2D, is consistent with the growing recognition of the long-term public health importance of supporting adequate prenatal nutrition. Diet is a broadly modifiable risk factor, and both maternal and paternal nutrition have an impact on the risk of metabolic syndrome, lipid dysregulation, fat deposition, obesity, and hypertension in offspring via a hypothesized mechanism of in utero epigenetic imprinting.^41–43^ Both epidemiological and animal studies highlight that undernutrition, overnutrition, and inadequate diet composition negatively impact fetoplacental growth and metabolic patterns, potentially having adverse later life metabolic effects in the offspring.^44^ Additionally, our data may also offer a window into the role by which nonnutritional factors affecting fetal growth, such as congenital heart disease and premature birth, may predispose affected individuals to long-term risk of cardiometabolic disease in adulthood.^45–47^

Our results indicate that some proportion of common chronic diseases of adulthood could potentially be reduced by achieving optimal fetal nutrition. Short-term follow-up of children born after randomized nutritional interventions in pregnancy describe beneficial effects on growth, vascular function, lipid levels, glucose tolerance, and insulin sensitivity, although longer-term studies examining nutrition and growth in premature infants display a more complex set of relationships.^48,49^ Considered in the context of populations, our data suggest that attention to prenatal nutrition and intrauterine growth may have long-term consequences regarding the risk of CAD, obesity, and diabetes mellitus in adult life.

### Strengths and Limitations

To our knowledge, this is the largest and most comprehensive study of associations of birthweight with outcome to date. Additionally, we used 3 different IVs to maximize power and to decrease risk of pleiotropy and several methods for MR analyses all yielding consistent effects for the tested hypotheses. However, our study is limited by the study samples of middle-aged to elderly individuals of European descent from a rich country. Hence, generalizability of our findings to other populations where the diet, prenatal care, prevalence, and predispositions of cardiometabolic disease are different is unknown. Further, although we excluded variants with higher likelihood of pleiotropy from our analysis and applied a range of sensitivity analyses and methods robust to pleiotropy, little is known about the mechanisms underlying loci included in the IV. Although our comprehensive analytic framework did not indicate any presence of horizontal pleiotropy, it is possible that some or all of these loci may also have a direct influence on the processes leading to CAD or T2D independent of intrauterine growth. In addition, despite the large sample in this study, statistical power to detect potentially causal relationships was limited for some traits, at least for the effect sizes from our observational analyses (in particular, DBP and WHR; Table). Finally, our design did not take into account maternal genetic variation, which may influence fetal growth indirectly through the intrauterine environment. Indeed, birthweight can be considered as the result of a developmental process started at conception and influenced by many factors during pregnancy, and future MR studies could be designed to consider both maternal and fetal genotypes as instruments for intrauterine exposures.

### Conclusions

In conclusion, we demonstrate that intrauterine growth restriction, as evidenced by lower birthweight, is causally related with increased susceptibility to T2D and CAD but that this effect is independent of adult hypertension or obesity, which has been previously hypothesized to be mediators of such an association. Our study supports the notion that population-level interventions improving prenatal nutrition and growth may improve cardiometabolic disease profiles later in life, but this needs to be confirmed using other study designs, such as large-scale community-based intervention trials, and MR analyses performed with both maternal and fetal genotypes as instruments.

## Acknowledgments

This research has been conducted using the UK Biobank Resource under application number 13721.

## Sources of Funding

This research was performed with support from National Institutes of Health (1R01HL135313-01). Dr Tikkanen was supported by the Finnish Cultural Foundation, Finnish Foundation for Cardiovascular Research, and Emil Aaltonen Foundation.

## Disclosures

None.

## Supplementary Material
