# Supplementary material for: Birthweight, Type 2 Diabetes Mellitus, and Cardiovascular Disease: Addressing the Barker Hypothesis With Mendelian Randomization
Source: Circ Genom Precis Med. 2018 Jun 6;11(6):e002054. doi: 10.1161/CIRCGEN.117.002054 (PMC6447084; doi:10.1161/CIRCGEN.117.002054)
Supplement: Supplementary file 1 [file hcg-11-e002054-s001.pdf]

SUPPLEMENTAL MATERIAL

Supplemental Methods 2
Study Sample 2
- **UK Biobank** 2
- **Definition of Exposure and Outcomes for Observational Analyses** 2
- **Definition of Confounders for Observational Analyses** 3
- **Data from GWAS Consortia** 3

Statistical Methods 4
- **Mendelian Randomization** 4

Supplemental Reference 6
Supplemental Tables 8
Supplemental Figures 16

Supplemental Methods

<u>Study Sample</u>

UK Biobank

We used the data collected at the UK Biobank (UKB) assessment centers at baseline, combined with the information on incident disease events from the hospital and death registries. Eligible for the present study were the 280,344 participants in UK Biobank that recalled their birth weight. From this study sample, we excluded 158 individuals who had withdrawn consent at the time of the study. Further, to focus on term pregnancies and linear effects of birth weight (BW), we excluded 42,555 individuals reporting a BW under 2.5 kg or over 4.5 kg, which is also consistent with the largest meta-analysis of BW to date, performed by the Early Growth Genetics (EGG) Consortium[1]. Consequently, we had a total of 237,631 eligible individuals for our observational analyses. In addition, for our Cox regression analyses, we excluded 17,717 individuals with prevalent cardiovascular disease (CVD; coronary artery disease [CAD], atrial fibrillation [AF], ischemic stroke [IS], hemorrhagic stroke or heart failure), as defined below. For the genome-wide association studies (GWAS) of SBP and DBP, we included all unrelated individuals with European ancestry that passed quality control and had a valid measurement of blood pressure (regardless of whether BW was assessed; N=337,229 and N= 337,235; respectively). The selection of unrelated individuals with European ancestry based on principal component analysis, and genetic markers with minor allele count ≥ 30 and an imputation quality metric $r^2 \geq 0.8$. Details of these measurements can be found in the study protocol[2] and in the UKB Data Showcase (http://biobank.ctsu.ox.ac.uk/crystal/).

Definition of Exposure and Outcomes for Observational Analyses

In our study, the exposure of interest was self-reported BW (field ID 20022).

Cardiovascular outcomes were defined using the in-patient hospital and death registries, including only primary causes to maximize specificity. CAD was defined as International Classification of Diseases (ICD) edition 9 codes 410-411, edition 10 codes I20.0, I21, and I22; and surgical codes for percutaneous transluminal coronary angioplasty and coronary artery bypass graft (codes K40-K46, K49-K50, and K75). AF was defined as ICD-9 code 427.3, ICD-10 code I48, and surgical codes K50.1, K62.2-K62.4). Stroke was defined as ischemic (ICD-9: 433-434, ICD-10: I63) or hemorrhagic stroke (ICD-9: 430-432, ICD-10: I60-I62). Heart failure

was defined as ICD-9 code 428 and ICD-10 code I50. The hospital registry-based follow-up ended on March 31, 2015 in England; August 31, 2014 in Scotland; and February 28, 2015 in Wales. Individuals were censored on these dates, time of event in question or the time of death, whichever occurred first. The death registry included all deaths that occurred before January 31, 2016 in England and Wales, and November 30, 2015 in Scotland. Cardiovascular death was defined using all of the above ICD-10 codes in the death registry. Type 2 diabetes (T2D) was defined as having a diagnosis of ICD-9 codes 250.10 or 250.12, or ICD-10 code E11 in the in-patient hospital register; being treated with anti-diabetic medication (metformin, glucophage, glibenclamide, daonil, gliclazide, diamicron, glipizide, minodiab, glibenese, glimepiride, amaryl, tolbutamide, acarbose, glucobay, repaglinide, nateglinide, starlix, pioglitazone, actos, insulin) or diabetes diagnosed by doctor (ID 2443).

Definition of Confounders for Observational Analyses

We used data from questionnaires to define age (field ID 21003), sex (ID 31), region of the UKB assessment center (ID 54; recoded to three countries: UK, Scotland and Wales), ethnicity (ID 21000; recoded to four groups: white, black, Asian, mixed), Townsend index reflecting socioeconomic status (ID 189), and maternal smoking status (ID 1789). Physical measurements were used to define body mass index (BMI) (ID 21001), systolic blood pressure (SBP) (ID 4080, but if missing ID 93), and diastolic blood pressure (DBP) (ID 4079, but if missing ID 94), body fat percentage (ID 23099), and waist-to-hip ratio (WHR) (ID 48/ID 49). Lipid medications (ID 20003; including following medications: simvastatin, pravastatin, fluvastatin, atorvastatin, rosuvastatin, ezetimibe, nicotinic acid product or fenofibrate) were used as a proxy for hyperlipidemia, as lipid measurements were not available in UKB at the time of the present study.

Data from GWAS Consortia

The genetic instruments for our Mendelian randomization (MR) analyses were different genetic risk scores combining independent single nucleotide polymorphisms (SNPs) associated with BW in the most recent GWAS at genome-wide significance (P< 5x10-8)[1]. Genetic variants associated with CAD, AF, IS and hypertension were extracted from the CARDIoGRAMplusC4D consortium[3], the Atrial Fibrillation Genetics (AFGen) Consortium[4], the International Stroke

Genetics Consortium (ISGC)[5] and the International Consortium for Blood Pressure (ICBP)[6], respectively. Results from the Genetic Investigation of ANthropometric Traits (GIANT) consortium were used to identify genetic variants associated with BMI[7] and WHR[8]. Genetic variants associated with high density lipoproteins (HDL), low density lipoprotein (LDL), and triglycerides (TG) were identified from GWAS data provided by the Global Lipids Genetic Consortium (GLGC)[9]. Additionally, genetic instruments for T2D were identified by means of the DIAbetes Genetics Replication and Meta-analysis (DIAGRAM) Consortium[10]. Finally, genetic variants associated with 2-hour post-challenge glucose[11], plasma levels of fasting glucose, and fasting insulin[12] were identified from publications by the Meta-Analysis of Glucose and Insulin related traits Consortium (MAGIC).

Statistical Methods

Mendelian Randomization

From the 59 lead variants showing genome-wide significance in the European meta-analysis of BW (EGG)[1], we excluded rs72851023 from inclusion in any of our instrumental variables (IVs). This variant is located in one of the most intensely studied imprinted gene clusters, the insulin-like growth factor 2 (IGF2) locus[1]. Previous studies have shown that the paternally expression of IGF2 promotes fetal growth, and it is significantly correlated with crown-rump length and BW[13].

We clustered variants based on their associations with outcomes and confounders to understand any larger patterns of underlying pleiotropic or mediating effects, by using hierarchical clustering with Euclidean distance and Ward method by means of the R software (http://www.R-project.org/)

We used publicly available GWAS summary statistics of CAD[3], AF[4], IS[5], SBP and DBP adjusted for BMI[6], BMI[14], WHR[8], HDL, LDL, TG[9], T2D[10], 2-hour glucose[11], fasting glucose, and fasting insulin[12] as outcomes. The numbers of variants included in the IVs differed for different outcomes. Some of the analyses were performed using data from Metabochip efforts with a lower number of variants in the GWAS summary statistics. Although this led to fewer variants in the IVs for these outcomes (Table 2), we choose these datasets since they were larger than any available full GWAS summary statistics, resulting in higher statistical power for the

MR analyses. Since the publically available GWAS of blood pressure was adjusted for BMI, we additionally conducted a GWAS of SBP and DBP without adjustment for BMI in the UKB data (N=337,229 and N= 337,235; respectively). In this analysis, we used a linear regression assuming additive model for association between phenotypes and genotype dosages by using PLINK (version 2.0)[15]. We included age, sex, batch and ten principal components as covariates.

We performed two-sample MR, which estimates the causal effect by contrasting the SNP effects on the exposure with the SNP effects on the outcome using independent datasets. If lead variants from EGG included in our IV were not available in the outcome summary statistics, we used the proxy with highest LD (at least $r^2 \geq 0.8$ for inclusion) defined using 1000 Genomes European data. The alleles from the exposure and outcome GWAS were harmonized with regards to effect alleles and strands.

For mediation MR analyses, we performed weighted linear regression models using the effect sizes of each GWAS significant SNP for BW (β_{BW}) and BMI (β_{BMI}) as predictor variables, and the effect sizes of CAD (β_{CAD}) or T2D (β_{T2D}) as the outcome variables, where the weights were defined by inverse standard errors of β_{CAD} or β_{T2D}[16,17].

Consistency of the causal estimates across all SNPs included in the IVs was evaluated with heterogeneity statistics, and Egger regression was used to assess directional horizontal pleiotropy.

Supplemental Reference

1. Horikoshi M, Beaumont RN, Day FR, Warrington NM, Kooijman MN, Fernandez-Tajes J, et al. Genome-wide association for birth weight and correlation with adult disease. Nature. 2016;538:248-252.
2. UK Biobank Coordinating Centre. UK Biobank: Protocol for a large-scale prospective epidemiological resource UK Biobank Coordinating Centre Stockport. *Design*. 2007;6:1–112.
3. Nikpay M, Goel A, Won HH, Hall LM, Willenborg C, Kanoni S, et al. A comprehensive 1000 Genomes-based genome-wide association meta-analysis of coronary artery disease. *Nat. Genet.* 2015;47:1121-1130.
4. Christophersen IE, Rienstra M, Roselli C, Yin X, Geelhoed B, Barnard J, et al. Large-scale analyses of common and rare variants identify 12 new loci associated with atrial fibrillation. *Nat Genet*. 2017;49:946–952.
5. Pulit SL, McArdle PF, Wong Q, Malik R, Gwinn K, Achterberg S, et al. The NINDS Stroke Genetics Network: a genome-wide association study of ischemic stroke and its subtypes. *Lancet. Neurol.* 2016; 15: 174–184.
6. Ehret GB, Ferreira T, Chasman DI, Jackson AU, Schmidt EM, Johnson T, et al. The genetics of blood pressure regulation and its target organs from association studies in 342,415 individuals. *Nat Genet*. 2016;48:1171-1184.
7. Locke AE, Kahali B, Berndt SI, Justice AE, Pers TH, Day FR, et al. Genetic studies of body mass index yield new insights for obesity biology. *Nature*. 2015;518:197–206.
8. Shungin D, Winkler TW, Croteau-Chonka DC, Ferreira T, Locke AE, Mägi R, et al. New genetic loci link adipose and insulin biology to body fat distribution. *Nature*. 2015;518:187–196.
9. Willer CJ, Schmidt EM, Sengupta S, Peloso GM, Gustafsson S, Kanoni S, et al. Discovery and refinement of loci associated with lipid levels. *Nat Genet*. 2013;45:1274–1283.
10. Morris AP, Voight BF, Teslovich TM, Ferreira T, Segrè A V, Steinthorsdottir V, et al. Large-scale association analysis provides insights into the genetic architecture and pathophysiology of type 2 diabetes. *Nat Genet*. 2012;44:981–990.
11. Scott RA, Lagou V, Welch RP, Wheeler E, Montasser ME, Luan J, et al. Large-scale association analyses identify new loci influencing glycemic traits and provide insight into the underlying biological pathways. *Nat Genet*. 2012;44:991–1005.
12. Manning AK, Hivert M-F, Scott RA, Grimsby JL, Bouatia-Naji N, Chen H, et al. A genome-wide approach accounting for body mass index identifies genetic variants influencing fasting glycemic traits and insulin resistance. *Nat Genet*. 2012;44:659–669.
13. Moore GE, Ishida M, Demetriou C, Al-Olabi L, Leon LJ, Thomas AC, et al. The role and interaction of imprinted genes in human fetal growth. *Philos Trans R Soc B Biol Sci*. 2015;370:20140074–20140074.
14. Berndt SI, Gustafsson S, Mägi R, Ganna A, Wheeler E, Feitosa MF, et al. Genome-wide meta-analysis identifies 11 new loci for anthropometric traits and provides insights into genetic architecture. *Nat Genet*. 2013;45:501-12
15. Chang CC, Chow CC, Tellier LC, Vattikuti S, Purcell SM, Lee JJ. Second-generation PLINK: rising to the challenge of larger and richer datasets. *Gigascience*. 2015;4:7.
16. Burgess S, Thompson SG. Multivariable Mendelian randomization: The use of pleiotropic genetic variants to estimate causal effects. *Am J Epidemiol*. 2015;181:251–260.

17. Do R, Willer CJ, Schmidt EM, Sengupta S, Gao C, Peloso GM, et al. Common variants associated with plasma triglycerides and risk for coronary artery disease. *Nat Genet*. 2013;45:1345–1352.

Supplemental Tables

Supplemental Table I. Baseline characteristics of UK Biobank participants of the present investigation of eligible samples reporting a birthweight (BW) between 2.5 and 4.5 kg (N=237,631) and non-eligible samples reporting missing data and low/high BW values (N=264,977).

	**Eligible Samples**	**Non-eligible samples**
**Variables**	**N (%) Mean (SD)**	**N (%) Mean (SD)**
Sex: Female	144,917 (61)	128,543 (48)
Sex: Males	92,734 (39)	136,434 (51)
Age	55.0 (8.1)	57.92 (7.8)
Ethnicity: White	230,059 (97)	242,752 (92)
Ethnicity: Black	1,835 (1)	6,230 (2)
Ethnicity: Asian	2,514 (1)	8,942 (4)
Ethnicity: Mixed	2,527 (1)	4,991 (2)
Maternal smoking *	59,659 (25)	66,996 (25)
BMI, kg/m^2	27.2 (4.8)	27.6 (4.8)
BW, kg	3.37 (0.42)	
T2D	9,839 (4)	17,271 (6)
Lipid Medications	31,245 (13)	51,122 (19)
SBP, mmHg	138.3 (19.5)	141.1 (19.8)
DBP, mmHg	82.0 (10.7)	82.5 (10.7)
Body Fat, %	31.8 (8.5)	31.2 (8.6)
WHR	0.86 (0.09)	0.88 (0.09)

Data is mean (standard deviation) or N (%). * Refers to whether mothers of the UK Biobank participants were smoking regularly around the time when they were born.
Abbreviations: SD, standard deviation; BMI, body mass index; BW, birthweight; T2D, type 2 diabetes; LIP, lipids medications; SBP, systolic blood pressure; DBP, diastolic blood pressure; WHR, waist-to-hip ratio.

Supplemental Table II. P-values of associations of birthweight (BW) variants with other traits in the UK Biobank data. Highlighted in red p-values of variants excluded from the main instrument variable (IV2) in the Mendelian randomization analysis.

SNP	BW	SBP	DBP	WHR	BF	BMI	LIP	T2D	CHD	AF	STR	MS	TOW
rs1042725	8.17E-08	1.29E-05	6.66E-01	1.05E-01	9.67E-01	2.29E-01	3.02E-02	2.46E-01	2.05E-01	4.21E-01	8.03E-01	9.40E-01	2.93E-01
SREBF2_rs10483213	2.29E-02	2.36E-01	6.11E-01	8.42E-01	3.26E-01	9.41E-02	7.69E-01	8.55E-01	7.84E-01	5.89E-01	8.87E-01	9.16E-01	3.10E-01
rs10818797	5.87E-04	5.72E-04	1.62E-01	1.84E-02	3.82E-02	3.95E-03	2.60E-01	5.69E-01	6.63E-01	2.41E-02	8.81E-01	5.68E-01	1.43E-01
MTNR1B_rs10830963	1.04E-04	1.25E-01	9.17E-01	3.39E-01	6.84E-01	1.90E-02	6.68E-03	1.96E-01	6.65E-01	5.78E-01	6.32E-01	9.23E-01	8.87E-02
rs10872678	1.73E-06	2.13E-01	9.82E-01	4.36E-03	3.26E-01	9.55E-01	5.72E-01	1.81E-01	1.21E-01	6.39E-01	2.79E-01	9.70E-01	5.02E-01
CPA3_rs10935733	5.22E-06	1.29E-01	3.08E-01	5.91E-01	2.49E-01	3.91E-01	1.50E-01	4.10E-01	3.10E-01	7.94E-02	4.49E-01	3.83E-01	2.01E-01
rs111778406	9.22E-06	4.97E-03	8.52E-02	1.28E-02	2.83E-01	3.79E-01	9.62E-02	7.58E-01	8.15E-01	1.08E-01	5.34E-01	9.77E-01	4.80E-01
CLDN7_rs113086489	3.10E-13	2.97E-03	2.75E-04	1.66E-02	1.14E-04	3.88E-03	1.89E-01	9.52E-01	6.83E-01	7.47E-01	9.81E-01	1.95E-01	3.48E-01
ADCY5_rs11719201	3.87E-11	3.35E-01	7.27E-01	4.11E-01	4.63E-01	3.59E-01	3.88E-01	1.86E-01	2.42E-01	2.30E-01	4.82E-01	5.95E-01	9.32E-03
IGF2BP3_rs11765649	3.84E-09	2.36E-01	5.34E-01	9.70E-01	4.84E-01	8.73E-01	7.75E-01	1.90E-01	7.43E-01	6.31E-01	9.55E-01	1.53E-03	1.40E-01
rs1187118	5.60E-07	6.17E-02	6.94E-01	7.64E-04	7.07E-01	2.84E-01	3.88E-02	8.71E-01	2.86E-04	9.50E-01	2.88E-01	3.53E-01	7.60E-02
SLC45A4_rs12543725	2.45E-04	4.22E-01	9.44E-01	1.89E-01	3.09E-01	2.90E-01	9.90E-01	7.75E-01	7.64E-01	5.65E-01	4.09E-01	4.78E-01	7.52E-01
FES_rs12906125	7.86E-07	5.75E-13	7.21E-10	2.87E-01	4.10E-01	2.19E-01	1.86E-04	8.74E-01	7.93E-08	7.59E-01	2.89E-01	2.01E-01	5.12E-01
ANK1-NKX6.3_rs13266210	1.58E-05	6.36E-01	6.69E-01	6.25E-01	2.75E-01	8.04E-02	5.96E-01	6.12E-01	3.57E-01	1.31E-01	9.00E-01	3.46E-01	4.95E-01
KREMEN1_rs134594	3.04E-06	6.81E-01	7.23E-01	5.62E-03	9.87E-01	3.97E-01	2.60E-01	1.60E-02	7.58E-01	9.35E-01	2.43E-02	1.93E-01	6.82E-01
HMGA2_rs1351394	3.77E-08	4.01E-06	5.96E-01	2.09E-01	3.58E-01	1.07E-01	5.50E-02	3.31E-01	3.13E-01	4.06E-01	8.02E-01	8.31E-01	2.92E-01
YKT6-GCK_rs138715366	4.43E-16	2.07E-01	7.62E-01	1.13E-01	2.45E-01	2.35E-01	5.48E-01	4.04E-01	5.35E-01	2.08E-01	5.65E-01	5.46E-02	5.36E-01
ABCC9_rs139975827	3.50E-03	4.26E-01	4.26E-01	6.28E-02	4.47E-02	8.40E-01	4.78E-01	3.99E-01	5.64E-01	4.51E-01	9.06E-02	4.47E-01	1.32E-01
rs1411424	3.62E-04	2.18E-02	4.82E-01	1.68E-04	7.73E-01	5.93E-01	7.73E-01	3.11E-01	1.87E-01	2.77E-01	3.85E-01	4.10E-01	6.29E-01
L3MBTL3_rs1415701	9.10E-03	2.01E-01	8.20E-01	1.79E-01	5.21E-01	6.29E-01	3.64E-01	7.61E-01	4.15E-02	9.18E-01	9.16E-02	6.34E-01	6.95E-01
SUZ12P1-CRLF3_rs144843919	4.08E-06	8.64E-01	3.06E-02	2.07E-01	1.29E-01	1.82E-01	1.05E-01	9.65E-01	3.98E-01	8.53E-01	3.76E-01	9.63E-01	8.49E-01
rs17034876	7.49E-19	1.73E-01	5.68E-02	2.15E-01	1.21E-01	8.98E-02	1.00E-01	9.24E-02	5.05E-01	2.85E-01	2.85E-01	9.42E-01	5.51E-01
rs1801253	1.28E-06	1.09E-04	6.27E-07	6.73E-01	3.50E-01	6.53E-01	9.32E-01	8.14E-01	3.62E-02	1.83E-01	5.44E-01	2.17E-01	4.56E-01
rs181211713	1.84E-03	4.46E-01	7.09E-01	4.68E-03	1.19E-01	8.07E-01	4.38E-02	8.09E-01	7.09E-01	1.16E-01	3.46E-01	9.86E-01	9.01E-01
RNF219-AS1_rs1819436	1.60E-03	5.19E-01	9.72E-01	9.87E-01	7.95E-01	5.05E-02	1.14E-01	7.78E-01	1.90E-01	8.71E-01	6.69E-02	8.31E-01	8.61E-01
rs2131354	6.65E-09	7.99E-01	3.05E-01	2.73E-03	3.64E-01	5.84E-01	3.23E-01	2.30E-01	7.39E-01	2.00E-01	9.86E-01	8.59E-01	2.73E-01
rs2168443	2.17E-04	1.89E-01	2.50E-01	8.45E-05	5.82E-01	7.08E-01	1.17E-03	6.70E-01	3.50E-02	8.95E-01	7.53E-01	4.57E-01	1.59E-01
NRIP1_rs2229742	2.33E-05	5.09E-04	4.48E-01	9.51E-02	3.97E-01	3.43E-01	1.84E-03	7.50E-01	2.82E-01	9.61E-01	2.59E-01	2.62E-02	1.87E-01
rs2306547	2.93E-05	6.33E-01	8.83E-01	4.04E-01	6.18E-01	8.34E-01	3.01E-01	8.32E-01	4.04E-01	4.13E-02	5.83E-01	2.04E-01	9.22E-01
PLEKHA1_rs2421016	7.46E-05	9.14E-02	6.67E-02	7.73E-01	1.22E-01	7.64E-02	8.90E-01	4.00E-01	4.25E-01	4.59E-01	1.41E-01	2.34E-01	4.68E-01
WNT4-ZBTB40_rs2473248	2.34E-01	8.83E-01	7.92E-01	3.46E-01	5.29E-01	5.66E-01	9.95E-01	7.06E-01	1.84E-01	7.61E-01	4.35E-01	9.28E-01	4.88E-01
rs2497304	7.06E-07	4.37E-03	3.78E-04	5.20E-01	6.84E-03	6.65E-03	8.22E-01	2.60E-01	9.80E-01	7.48E-01	1.90E-01	4.80E-01	3.81E-01
rs2724475	1.83E-05	1.89E-03	3.11E-01	4.91E-01	3.01E-01	3.34E-01	3.79E-03	4.14E-01	3.59E-02	4.59E-01	2.60E-01	9.49E-01	5.23E-01
C20orf203_rs28530618	2.80E-03	2.07E-01	6.03E-01	4.15E-01	8.12E-01	4.26E-01	3.94E-02	9.68E-01	2.66E-02	6.83E-01	6.36E-01	9.76E-01	6.32E-01
rs2946179	1.50E-02	1.34E-02	2.10E-01	1.36E-02	1.71E-04	2.95E-01	1.19E-01	3.50E-01	6.89E-02	1.84E-02	2.35E-01	3.90E-01	2.95E-01
rs34217484	1.03E-04	7.06E-01	8.03E-01	9.71E-01	1.84E-01	3.27E-01	7.58E-01	4.86E-01	7.23E-01	9.86E-01	8.47E-01	5.39E-01	2.12E-01
CDKAL1_rs35261542	7.96E-09	7.82E-02	2.66E-01	9.49E-01	9.45E-02	2.44E-03	4.15E-03	3.42E-02	2.60E-01	9.76E-01	2.84E-01	5.81E-01	1.52E-01
ZBTB7B_rs3753639	3.20E-06	4.86E-01	7.25E-01	4.46E-03	7.67E-01	2.44E-04	7.44E-01	9.33E-01	8.09E-01	4.02E-02	8.49E-01	4.22E-01	3.52E-01
rs3780573	5.22E-08	2.11E-01	6.30E-01	3.40E-03	5.04E-03	4.74E-01	6.27E-01	8.43E-01	5.96E-01	6.26E-01	6.97E-01	1.94E-01	6.96E-01
rs4432842	7.73E-04	2.23E-01	2.76E-01	8.28E-01	8.65E-01	6.85E-01	2.27E-01	9.41E-01	5.05E-01	7.66E-01	1.34E-01	9.79E-01	4.34E-01
rs4836833	2.39E-06	1.80E-01	8.78E-02	7.78E-02	2.19E-02	2.53E-01	4.04E-01	5.82E-01	7.35E-01	8.34E-01	2.68E-01	5.58E-02	5.61E-01

MAFB_rs6016377	4.77E-04	4.04E-02	2.11E-02	4.57E-01	2.61E-03	1.57E-03	6.16E-04	3.08E-01	1.26E-02	4.57E-01	3.86E-02	3.09E-01	1.57E-01
JAG1_rs6040076	2.12E-07	1.56E-04	1.69E-04	2.56E-02	8.41E-02	5.43E-01	1.06E-01	7.65E-01	3.14E-01	6.59E-01	3.89E-01	2.71E-01	6.77E-01
rs6931514	2.73E-08	5.33E-02	3.10E-01	6.80E-01	7.20E-02	1.69E-03	3.46E-03	2.58E-02	2.42E-01	9.67E-01	3.18E-01	5.21E-01	2.39E-01
TBX20_rs6959887	1.94E-02	5.18E-01	4.63E-02	1.49E-01	7.26E-01	1.78E-01	5.02E-02	9.06E-01	3.00E-02	9.00E-01	7.00E-02	2.40E-01	8.28E-01
rs724577	2.28E-05	2.60E-03	3.58E-01	4.74E-01	3.31E-01	3.88E-01	3.06E-03	4.36E-01	4.01E-02	4.29E-01	2.29E-01	9.71E-01	4.99E-01
FCGR2B_rs72480273	1.33E-04	2.98E-01	3.04E-01	5.53E-01	4.94E-01	4.61E-02	4.05E-02	8.94E-01	7.75E-01	8.24E-01	5.45E-01	6.35E-01	5.61E-01
rs72833480	5.20E-02	5.26E-01	5.35E-04	3.25E-01	4.97E-02	4.57E-01	5.14E-02	8.35E-02	1.34E-02	9.01E-01	4.38E-01	8.16E-01	8.20E-01
INS-IGF2_rs72851023	4.12E-08	1.67E-02	8.86E-02	3.98E-01	8.33E-01	1.54E-01	9.54E-01	9.09E-01	2.98E-01	8.63E-01	9.12E-01	2.58E-01	5.06E-01
IGF1R_rs7402982	1.79E-06	2.16E-01	8.45E-01	3.18E-02	2.99E-01	9.80E-01	4.45E-01	6.56E-01	7.04E-01	8.38E-01	6.59E-01	1.06E-01	1.09E-01
rs740746	1.75E-06	1.24E-04	5.32E-07	9.14E-01	3.57E-01	6.59E-01	8.63E-01	9.89E-01	3.52E-02	2.14E-01	5.94E-01	2.37E-01	4.95E-01
rs79237883	1.06E-06	2.84E-15	3.65E-06	2.41E-02	1.54E-02	5.40E-05	8.19E-02	1.09E-02	2.31E-01	3.34E-01	2.03E-01	5.34E-02	8.81E-01
IGF1_rs7964361	1.44E-05	6.39E-01	9.73E-01	5.91E-01	7.44E-01	9.95E-01	7.19E-01	9.51E-01	3.34E-01	8.90E-01	9.83E-01	7.80E-01	2.38E-01
rs798498	8.34E-06	1.23E-01	1.10E-01	4.24E-01	4.54E-03	5.91E-01	9.13E-02	5.40E-01	7.01E-01	1.98E-01	7.74E-01	3.07E-01	9.41E-01
rs7998537	1.20E-03	7.36E-01	1.57E-01	9.95E-01	7.77E-01	1.77E-01	5.33E-01	6.98E-01	3.50E-01	7.25E-03	3.61E-01	7.42E-01	8.24E-01
5q11.2_rs854037	1.15E-03	9.48E-02	9.21E-01	5.58E-01	5.77E-01	4.58E-01	1.15E-01	3.33E-01	8.37E-01	7.74E-01	1.11E-01	7.62E-01	2.78E-01
rs900399	2.46E-18	2.08E-01	4.65E-01	1.04E-10	5.70E-02	1.72E-02	3.80E-03	1.91E-01	9.68E-01	1.76E-02	2.27E-01	9.39E-01	4.76E-01
rs900400	2.61E-18	1.96E-01	4.52E-01	1.14E-10	5.87E-02	1.72E-02	4.12E-03	1.89E-01	9.75E-01	1.50E-02	2.39E-01	9.22E-01	4.56E-01
rs9883204	1.08E-09	3.66E-01	6.81E-01	3.73E-01	4.37E-01	3.70E-01	5.32E-01	2.62E-01	4.24E-01	2.60E-01	9.92E-01	5.17E-01	4.03E-02

Variants associated with outcomes and confounders at GWAS significance; or with any of the outcomes or confounders at a P-value lower than the P-value for association with BW (highlighted in red) were excluded from the IV2 in the Mendelian randomization analysis.
Abbreviations: SBP, systolic blood pressure; DBP, diastolic blood pressure; WHR, waist-to-hip ratio; BF, body fat percentage; BMI, body mass index; LIP, lipids medications; T2D, type 2 diabetes; CAD, coronary artery disease; AF, atrial fibrillation; IS, ischemic stroke; MS, maternal smoking; TOW, Townsend index.

Supplemental Table III. Observational associations of birth weight with cardiovascular outcomes in UK Biobank using multivariable-adjusted linear and logistic regression, and multivariable-adjusted Cox proportional hazards models. **a.** Continuous outcomes: systolic and diastolic blood pressure (SBP, and DBP; respectively), body mass index (BMI), body fat percentage (BF), waist-to-hip ratio (WHR). **b.** Binary outcomes: coronary artery disease (CAD), atrial fibrillation (AF), ischemic stroke (IS), hemorrhagic stroke (HS), heart failure (HF), type 2 diabetes (T2D) and lipid medications (LIP).

a.

**Outcome**	**Beta**	**95 % CI**	**P**
SBP	-0.830	-0.750, -0.900	**<2e-16**
DBP	-0.263	-0.218, -0.308	**<2e-16**
BMI	0.041	0.037, 0.044	**<2e-16**
BF	0.018	0.015, 0.020	**<2e-16**
WHR	0.003	-0.001, 0.006	1.14E-01

b.

**Outcome**	**N**	**HR/OR**	**95 % CI**	**P**
CAD	2,656	0.854	0.779, 0.936	**1.34E-03**
AF	1,580	1.179	1.049, 1.326	8.88E-03
IS	688	0.881	0.735, 1.055	1.83E-01
HS	363	0.817	0.636, 1.049	1.43E-01
HF	255	1.059	0.790, 1.420	7.01E-01
T2D		0.832	0.790, 0.877	**1.51E-11**
LIP		0.839	0.812, 0.866	**<2e-16**

Estimates are from multivariable-adjusted linear (continuous risk factors), Cox proportional hazards models (cardiovascular outcomes) or logistic (lipid treatment and T2D) regression. The betas from linear regression represent SD change in outcome variable per SD change in BW, except for SBP and DBP where they represent the outcome in raw unit (mmHg) per SD change in BW. The HRs or ORs (Cox or logistic models) are reported per SD change in outcome and BW. All models are adjusted for age, sex, region of the UKB assessment center, ethnicity, maternal smoking and Townsend index. P-values significant after Bonferroni correction (taking all phenotypes into account; $P<0.004$) are bold. N, incident events in the UK Biobank.

Abbreviations: HR, hazard ratio; OR, odds ratio; CI, confidence interval.

Supplemental Table IV. Mendelian randomization analyses of associations of birthweight with different cardiovascular outcomes and risk factors, and type 2 diabetes.

	**IV1**	**N=57**			**IV2**	**N=45**			**IV3**	**N=36**		
**CAD**	**OR**	**95% CI**	**p**	**het**	**OR**	**95% CI**	**p**	**het**	**OR**	**95% CI**	**p**	**het**
IVW	0.71	(0.61, 0.82)	**2.77E-06**	2.50E-10	0.69	(0.60, 0.80)	**7.49E-07**	9.16E-05	0.76	(0.67, 0.87)	**5.57E-05**	0.092
Penalized Robust IVW	0.71	(0.64, 0.78)	**<1.00E-04**		0.66	(0.59, 0.74)	**<1.00E-04**		0.74	(0.66, 0.82)	**<1.00E-04**	
MR Egger	0.60	(0.39, 0.94)	2.82E-02	1.05E-10	0.51	(0.33, 0.79)	4.59E-03	1.10E-04	0.52	(0.37, 0.75)	**1.19E-03**	0.164
Weighted median	0.74	(0.65, 0.85)	**6.95E-05**		0.63	(0.54, 0.74)	**2.05E-08**		0.68	(0.58, 0.80)	**1.01E-05**	
	**IV1**	**N=49**			**IV2**	**N=39**			**IV3**	**N=38**		
**AF**	**OR**	**95% CI**	**p**	**het**	**OR**	**95% CI**	**p**	**het**	**OR**	**95% CI**	**p**	**het**
IVW	1.13	(0.98, 1.32)	1.02E-01	1.65E-02	1.15	(0.95, 1.39)	1.56E-01	2.35E-03	1.19	(1.01, 1.41)	3.57E-02	8.90E-02
Penalized Robust IVW	1.17	(1.01, 1.35)	4.30E-02		1.20	(1.04, 1.40)	6.40E-02		1.23	(1.01, 1.49)	4.90E-02	
MR Egger	1.48	(0.90, 2.45)	1.32E-01	1.37E-02	1.70	(0.87, 3.31)	1.26E-01	2.06E-03	1.43	(0.79, 2.59)	2.40E-01	6.22E-02
Weighted median	1.08	(0.89, 1.31)	4.77E-01		1.16	(0.95, 1.45)	2.07E-01		1.17	(0.93, 1.46)	1.77E-01	
	**IV1**	**N=57**			**IV2**	**N=45**			**IV3**			
**IS**	**OR**	**95% CI**	**p**	**het**	**OR**	**95% CI**	**p**	**het**				
IVW	0.84	(0.70, 1.01)	6.30E-02	1.70E-02	0.82	(0.67, 1.01)	6.50E-02	7.96E-02				
Penalized Robust IVW	0.82	(0.69, 0.97)	2.40E-02		0.78	(0.65, 0.93)	8.00E-03					
MR Egger	1.14	(0.63, 2.09)	6.63E-01	1.41E-02	0.92	(0.46, 1.84)	8.04E-01	5.29E-02				
Weighted median	0.75	(0.59, 0.95)	1.60E-02		0.70	(0.54, 0.91)	8.00E-03					
	**IV1**	**N=43**			**IV2**	**N=33**			**IV3**	**N=21**		
**SBP UKB**	**beta**	**se**	**p**	**het**	**beta**	**se**	**p**	**het**	**beta**	**se**	**p**	**het**
IVW	-0.28	0.80	7.23E-01	3.76E-66	-0.33	0.68	6.23E-01	6.29E-23	-0.43	0.43	3.20E-01	1.94E-01
Penalized Robust IVW	-0.725	0.53	1.75E-01		-0.41	0.474	3.98E-01		-0.41	0.47	3.86E-01	
MR Egger	1.19	2.24	5.97E-01	1.06E-67	-0.23	1.85	9.02E-01	2.15E-24	-0.30	1.02	7.72E-01	1.17E-01
Weighted median	0.22	0.54	6.78E-01		-0.09	0.56	8.68E-01		-0.36	0.58	5.30E-01	
	**IV1**	**N=43**			**IV2**	**N=33**			**IV3**	**N=24**		
**DBP UKB**	**beta**	**se**	**p**	**het**	**beta**	**se**	**p**	**het**	**beta**	**se**	**p**	**het**
IVW	0.44	0.41	2.93E-01	2.71E-51	0.21	0.34	5.43E-01	4.58E-15	-0.02	0.24	9.26E-01	9.51E-02
Penalized Robust IVW	0.21	0.23	3.78E-01		0.14	0.26	6.01E-01		-0.04	0.26	8.83E-01	
MR Egger	1.16	1.16	3.17E-01	1.08E-52	0.94	0.91	3.01E-01	1.40E-15	0.89	0.54	1.00E-01	1.40E-01
Weighted median	0.49	0.26	5.60E-02		0.40	0.29	1.72E-01		0.16	0.30	5.95E-01	
	**IV1**	**N=38**			**IV2**	**N=34**			**IV3**	**N=31**		
**SBP ICBP**	**beta**	**se**	**p**	**het**	**beta**	**se**	**p**	**het**	**beta**	**se**	**p**	**het**
IVW	-0.04	0.88	9.65E-01	1.72E-35	-1.04	0.54	5.34E-02	3.15E-05	-0.95	0.43	2.59E-02	1.11E-01
Penalized Robust IVW	-0.57	0.54	2.38E-01		-0.69	0.44	1.27E-01		-0.64	0.43	1.50E-01	
MR Egger	1.73	3.80	6.52E-01	5.61E-37	0.50	2.36	8.33E-01	1.34E-05	1.23	1.79	4.95E-01	1.03E-01
Weighted median	-0.46	0.57	4.14E-01		-0.77	0.57	1.74E-01		-0.76	0.55	1.70E-01	
	**IV1**	**N=38**			**IV2**	**N=34**			**IV3**	**N=33**		
**DBP ICBP**	**beta**	**se**	**p**	**het**	**beta**	**se**	**p**	**het**	**beta**	**se**	**p**	**het**
IVW	0.17	0.49	7.32E-01	3.42E-27	-0.55	0.28	4.99E-02	7.94E-03	-0.69	0.25	5.98E-03	1.15E-01
Penalized Robust IVW	-0.60	0.48	1.15E-01		-0.69	0.33	4.10E-02		-0.71	0.31	3.00E-02	

MR Egger	1.35	2.11	5.26E-01	2.57E-28	0.32	1.23	7.98E-01	4.71E-03	0.89	1.07	4.12E-01	1.25E-01
Weighted median	0.06	0.34	8.50E-01		-0.15	0.35	6.65E-01		-0.36	0.34	2.88E-01	
	**IV1**	**N=49**			**IV2**	**N=38**			**IV3**	**N=31**		
**BMI**	**beta**	**se**	**p**	**het**	**beta**	**se**	**p**	**het**	**beta**	**se**	**p**	**het**
IVW	0.11	0.02	**3.95E-07**	3.36E-04	0.13	0.03	**1.32E-06**	3.94E-04	0.06	0.03	3.66E-02	8.46E-02
Penalized Robust IVW	0.12	0.02	**<1.00E-04**		0.02	0.09	**<1.00E-04**		0.07	0.03	2.30E-02	
MR Egger	0.25	0.07	**1.50E-03**	7.70E-04	0.32	0.09	**1.28E-03**	1.55E-03	0.15	0.10	1.48E-01	6.50E-02
Weighted median	0.12	0.03	**9.86E-06**		0.18	0.03	**4.94E-09**		0.07	0.04	6.51E-02	
	**IV1**	**N=49**			**IV2**	**N=38**			**IV3**	**N=30**		
**WHR**	**beta**	**se**	**p**	**het**	**beta**	**se**	**p**	**het**	**beta**	**se**	**p**	**het**
IVW	0.01	0.04	7.56E-01	2.35E-22	-0.08	0.04	2.20E-02	5.19E-07	-0.03	0.03	3.57E-01	7.42E-02
Penalized Robust IVW	-0.01	0.03	8.45E-01		-0.07	0.03	1.50E-02		-0.03	0.03	2.51E-01	
MR Egger	0.16	0.14	2.53E-01	1.67E-22	-0.21	0.13	1.03E-01	3.55E-07	-0.13	0.11	2.36E-01	5.79E-02
Weighted median	0.00	0.03	1.00E+00		-0.08	0.04	2.77E-02		-0.01	0.04	7.69E-01	
	**IV1**	**N=50**			**IV2**	**N=38**			**IV3**	**N=35**		
**HDL**	**beta**	**se**	**p**	**het**	**beta**	**se**	**p**	**het**	**beta**	**se**	**p**	**het**
IVW	-0.05	0.04	2.69E-01	3.82E-20	-0.05	0.04	1.87E-01	2.74E-06	0.02	0.03	3.99E-01	3.31E-01
Penalized Robust IVW	0.03	0.02	1.60E-01		0.01	0.02	5.46E-01		0.02	0.03	3.26E-01	
MR Egger	-0.46	0.13	**9.99E-04**	5.46E-15	-0.32	0.13	1.88E-02	2.08E-05	-0.13	0.09	1.66E-01	3.75E-01
Weighted median	-0.02	0.04	6.58E-01		-0.02	0.04	6.58E-01		-0.01	0.04	7.42E-01	
	**IV1**	**N=50**			**IV2**	**N=38**			**IV3**	**N=37**		
**LDL**	**beta**	**se**	**p**	**het**	**beta**	**se**	**p**	**het**	**beta**	**se**	**p**	**het**
IVW	-0.07	0.03	9.89E-03	1.16E-02	-0.11	0.03	**4.09E-04**	2.56E-02	-0.10	0.03	**2.38E-04**	2.95E-01
Penalized Robust IVW	-0.05	0.03	7.00E-02		-0.11	0.03	**3.00E-03**		-0.10	0.03	5.00E-03	
MR Egger	-0.02	0.10	8.66E-01	7.36E-03	-0.01	0.12	9.19E-01	1.86E-02	-0.06	0.10	5.60E-01	2.24E-01
Weighted median	-0.03	0.04	4.08E-01		-0.10	0.04	1.96E-02		-0.10	0.04	2.04E-02	
	**IV1**	**N=49**			**IV2**	**N=37**			**IV3**	**N=**		
**TG**	**beta**	**se**	**p**	**het**	**beta**	**se**	**p**	**het**	**beta**	**se**	**p**	**het**
IVW	-0.03	0.03	3.41E-01	4.57E-07	-0.05	0.03	1.33E-01	5.64E-03	-0.02	0.03	5.84E-01	1.41E-01
Penalized Robust IVW	-0.05	0.03	8.60E-02		-0.04	0.03	1.90E-01		-0.02	0.03	6.02E-01	
MR Egger	0.13	0.11	2.39E-01	7.46E-07	-0.08	0.11	4.72E-01	2.80E-03	-0.02	0.11	8.63E-01	9.15E-02
Weighted median	-0.02	0.04	5.60E-01		-0.08	0.04	5.35E-02		-0.02	0.04	5.61E-01	
	**IV1**	**N=25**			**IV2**	**N=17**			**IV3**	**N=11**		
**T2D**	**OR**	**95% CI**	**p**	**het**	**OR**	**95% CI**	**p**	**het**	**OR**	**95% CI**	**p**	**het**
IVW	0.27	(0.17, 0.42)	**6.84E-09**	2.40E-26	0.19	(0.11, 0.34)	**1.77E-08**	1.35E-21	0.44	(0.33, 0.59)	**3.47E-08**	1.79E-01
Penalized Robust IVW	0.37	(0.31, 0.44)	**<1.00E-04**		0.25	(0.19, 0.34)	**<1.00E-04**		0.43	(0.33, 0.57)	**<1.00E-04**	
MR Egger	0.17	(0.03, 0.90)	4.79E-02	7.68E-28	0.12	(0.01, 1.27)	9.77E-02	1.52E-23	0.21	(0.06, 0.76)	4.10E-02	1.45E-01
Weighted median	0.35	(0.26, 0.47)	**2.10E-11**		0.33	(0.22, 0.48)	**1.38E-09**		0.35	(0.25, 0.49)	**4.29E-10**	
	**IV1**	**N=20**			**IV2**	**N=17**			**IV3**	**N=15**		
**2hr glucose**	**beta**	**se**	**p**	**het**	**beta**	**se**	**p**	**het**	**beta**	**se**	**p**	**het**
IVW	-0.53	0.16	**9.73E-04**	1.14E-08	-0.66	0.17	**9.54E-05**	1.15E-06	-0.42	0.10	**6.43E-05**	3.22E-01
Penalized Robust IVW	-0.48	0.09	**<1.00E-04**		-0.49	0.09	**<1.00E-04**		-0.48	0.07	**<1.00E-04**	
MR Egger	-1.01	0.62	1.21E-01	3.02E-09	-1.79	0.63	1.30E-02	9.82E-06	-1.07	0.38	1.37E-02	3.98E-01
Weighted median	-0.47	0.12	**1.33E-04**		-0.53	0.13	**7.23E-05**		-0.48	0.12	**1.09E-04**	

	**IV1**	**N=49**			**IV2**	**N=38**			**IV3**	**N=30**		
**Fasting glucose**	**beta**	**se**	**p**	**het**	**beta**	**se**	**p**	**het**	**beta**	**se**	**p**	**het**
IVW	-0.12	0.05	3.30E-02	2.11E-84	-0.14	0.07	4.85E-02	3.81E-87	0.00	0.03	8.62E-01	7.52E-02
Penalized Robust IVW	-0.07	0.02	**1.00E-03**		-0.10	0.03	**2.00E-03**		0.00	0.03	9.74E-01	
MR Egger	-0.07	0.19	7.16E-01	4.60E-87	-0.15	0.26	5.86E-01	1.10E-90	-0.02	0.10	8.34E-01	4.43E-02
Weighted median	-0.07	0.03	2.31E-02		-0.10	0.03	5.64E-03		-0.04	0.04	2.84E-01	
	**IV1**	**N=49**			**IV2**	**N=38**			**IV3**	**N=36**		
**Fasting insulin**	**beta**	**se**	**p**	**het**	**beta**	**se**	**p**	**het**	**beta**	**se**	**p**	**het**
IVW	-0.02	0.02	3.18E-01	7.06E-03	-0.01	0.02	7.84E-01	6.40E-03	-0.01	0.02	6.67E-01	1.28E-01
Penalized Robust IVW	-0.03	0.02	8.60E-02		-0.02	0.02	4.68E-01		-0.02	0.02	3.87E-01	
MR Egger	0.06	0.07	4.26E-01	5.99E-03	0.16	0.09	7.74E-02	1.33E-02	0.12	0.08	1.45E-01	1.53E-01
Weighted median	-0.03	0.03	2.37E-01		-0.01	0.03	6.60E-01		-0.01	0.03	6.22E-01	

Four separate methods were used to estimate causal effects of birthweight with different cardiovascular outcomes and risk factors, and type 2 diabetes: the standard inverse-variance weighted (IVW) regression, the robust penalized IVW; and two robust regression methods, the weighted median-based method, and Egger regression. We used three different instrument variables (IVs): IV1. Including all the 58 independent led variants from the GWAS for BW (to maximize statistical power); IV2. Excluding SNPs associated with CAD, AF, IS and T2D at GWAS significance, any confounders at GWAS significance; or with any of the confounders and CAD, AF, IS and T2D at a P-value lower than the P-value for association with BW (main model, combining retained power with low risk of pleiotropy); IV3. Excluding any variants showing significant heterogeneity (to further decrease risk of pleiotropy). P-values significant after Bonferroni correction (adjusting for the number of phenotypes, but not all models due to high correlation; P<0.003) are bold. All effects (beta or OR) are given in original units as provided by the consortia.

Abbreviations: CAD, coronary artery disease; AF, atrial fibrillation; IS, ischemic stroke; SBP, systolic blood pressure; DBP, diastolic blood pressure; UKB, UK Biobank; ICBP, International Consortium of Blood Pressure; BMI, body mass index; WHR, waist-to-hip ratio; HDL, high density lipoprotein; LDL, low density lipoproteins; TG, triglycerides; T2D, type 2 diabetes; N, number of SNPs; se, standard error, OR, odd ratio; CI, confidence interval; P, P-value.

Supplemental Table V. Association of birthweight (BW) and body mass index (BMI) with coronary artery disease (CAD) or type 2 diabetes (T2D) from mediation MR analyses to address whether BW had a causal effect on CAD and T2D independently of BMI.

**CAD**	**Beta**	**se**	**p**
BW	-0.280	0.076	3.44E-04
BMI	0.389	0.075	9.25E-07
**T2D**	**Beta**	**se**	**p**
BW	-0.958	0.320	0.004
BMI	0.530	0.246	0.034

Estimates are from multivariable MR weighted regression-based model.
Abbreviations: se, standard error; p, P-Value.

Supplemental Figures

Supplemental Figure I. Directed acyclic graph illustrating potential confounders (red) and mediators (blue) of the associations of birthweight (BW) with cardiovascular diseases (CVD).

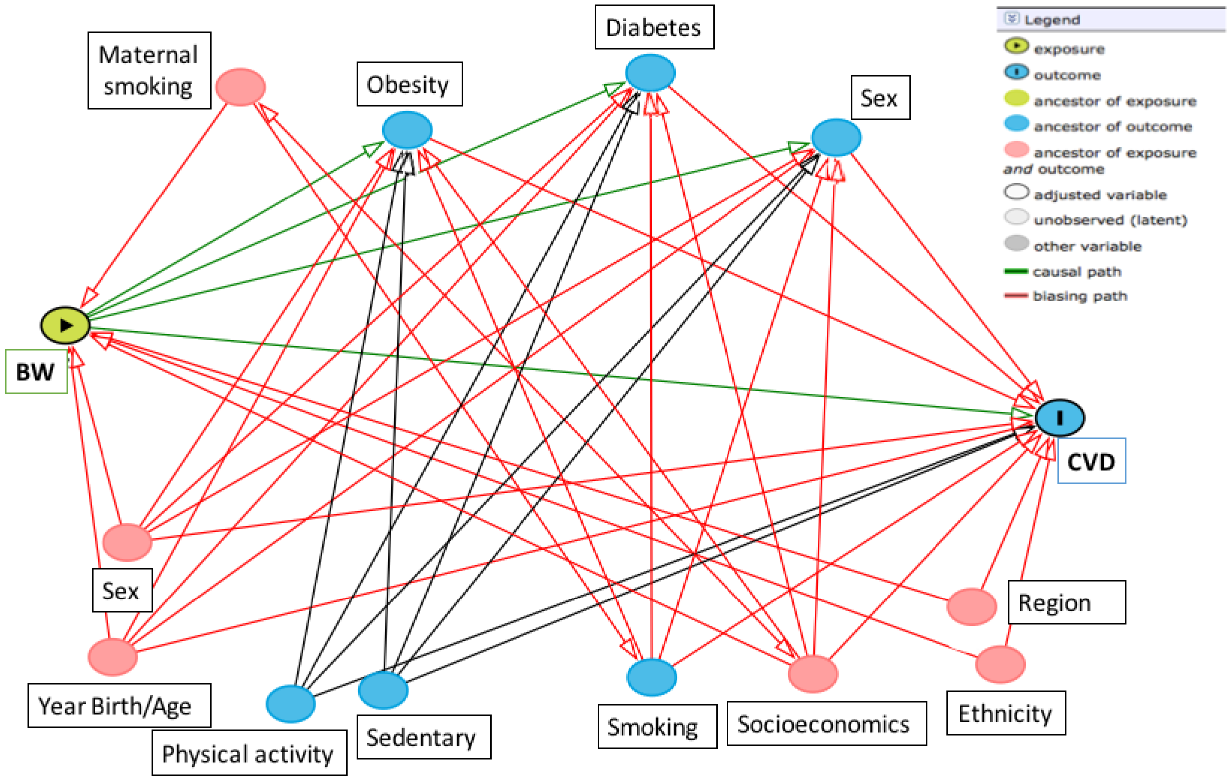

Supplemental Figure II. Hierarchical clustering of associations of birthweight (BW) loci with other traits in the UK Biobank data.

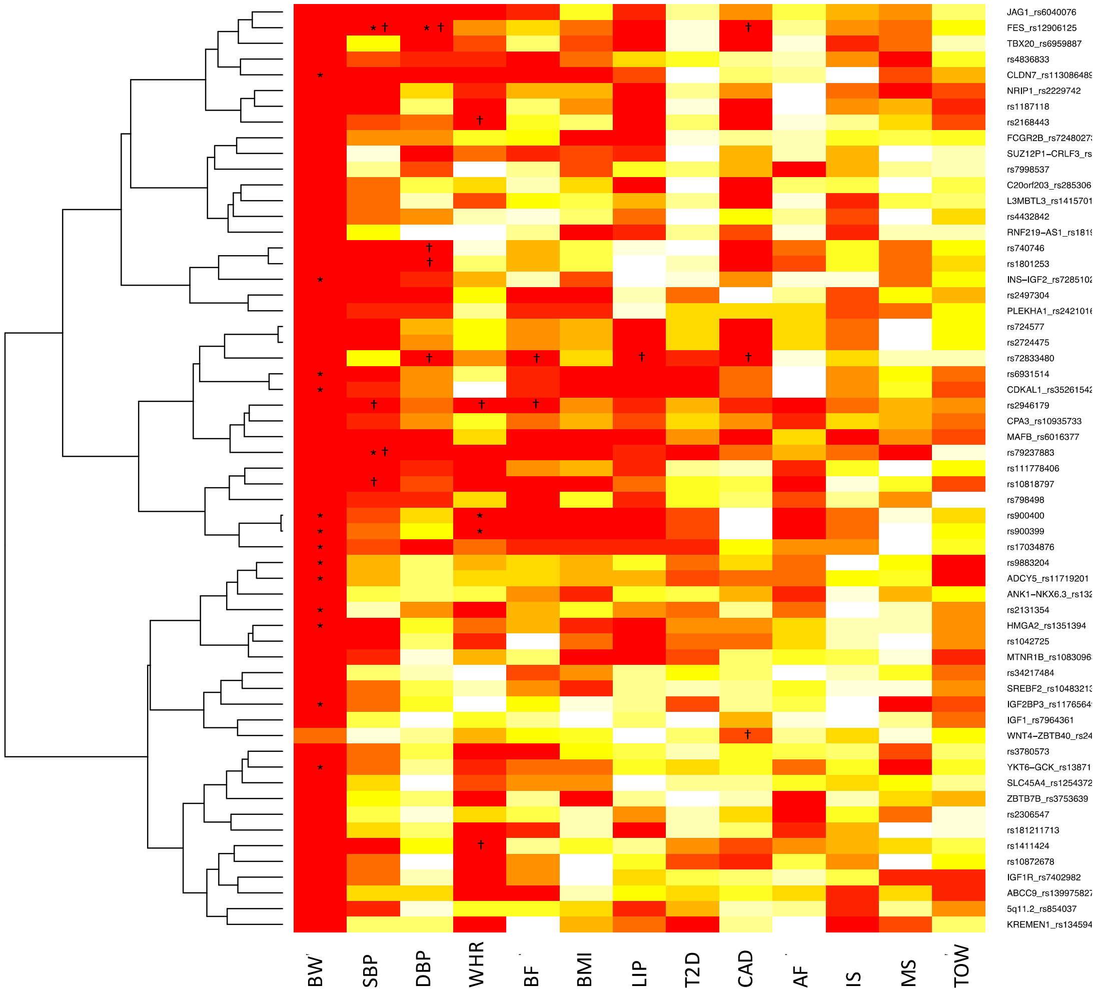

* denotes genome-wide significant ($P \leq 5x10^{-8}$) associations.

† denotes an association with a P-value lower than the P-value for association with BW.

Abbreviations: SBP, systolic blood pressure; DBP, diastolic blood pressure; WHR, waist-to-hip ratio; BF, body fat percentage; BMI, body mass index; LIP, lipids medications; T2D, type 2 diabetes; CAD, coronary artery disease; AF, atrial fibrillation; IS, ischemic stroke; MS, maternal smoking; TOW, Townsend index.

Supplemental Figure III. Effects of genetic variants on birthweight (BW) and risk of **a.** coronary artery disease (CAD); **b.** atrial fibrillation (AF); **c.** ischemic stroke (IS); **d.** systolic blood pressure in UK Biobank (SBP UKB); **e.** diastolic blood pressure in UK Biobank (DBP UKB); **f.** systolic blood pressure from International Consortium of Blood Pressure (SBP); **g.** diastolic blood pressure from International Consortium of Blood Pressure (DBP); **h.** body mass index (BMI); **j.** waist-to-hip ratio (WHR); **k.** high density lipoprotein (HDL); **i.** low density lipoproteins (LDL); **l.** triglycerides (TG); **m.** type 2 diabetes (T2D); **n.** 2-hour glucose; **o.** fasting glucose; **p.** fasting insulin, using the IV2 with 46 variants. Variants showing significant heterogeneity are highlighted in red.

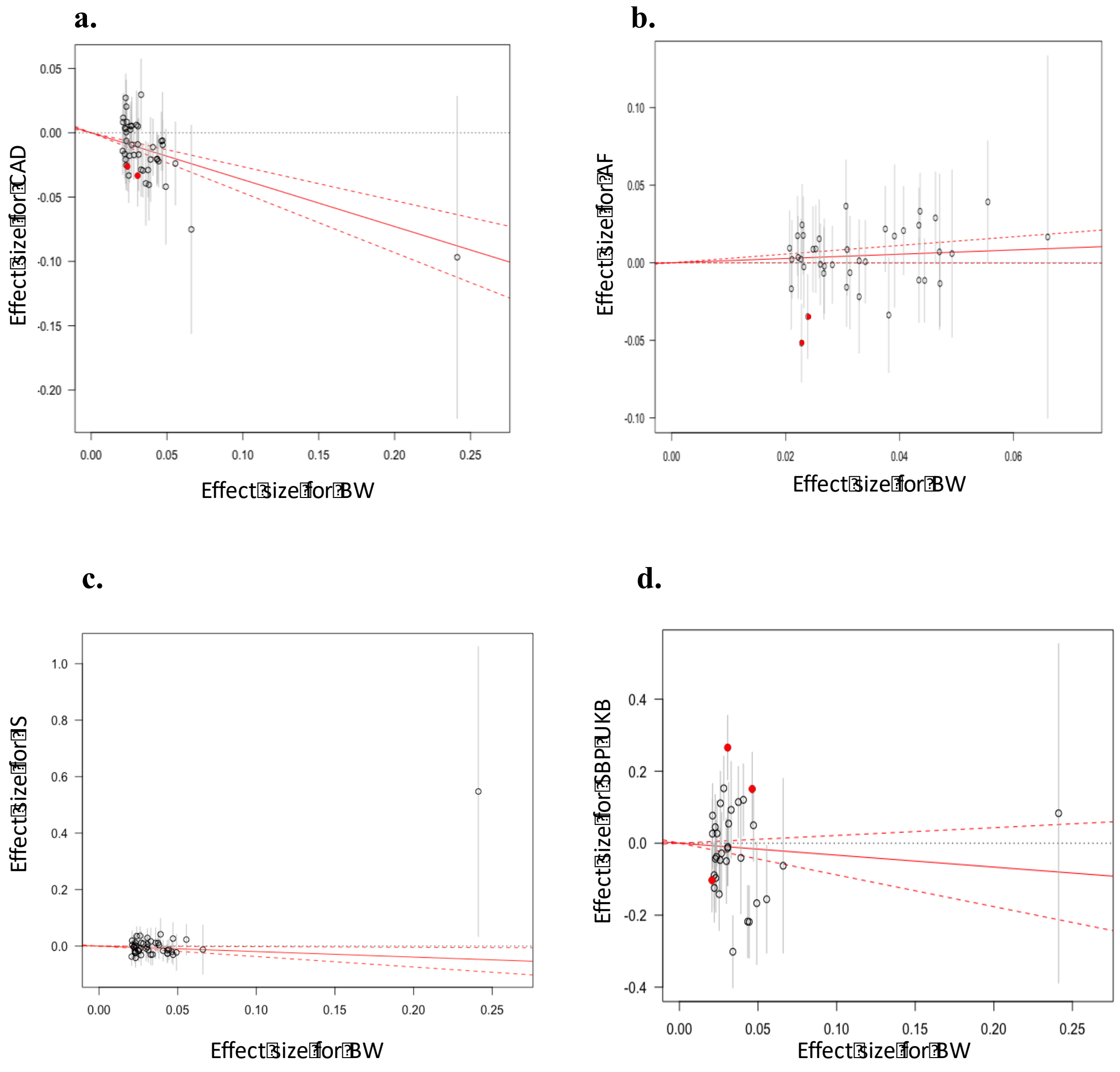

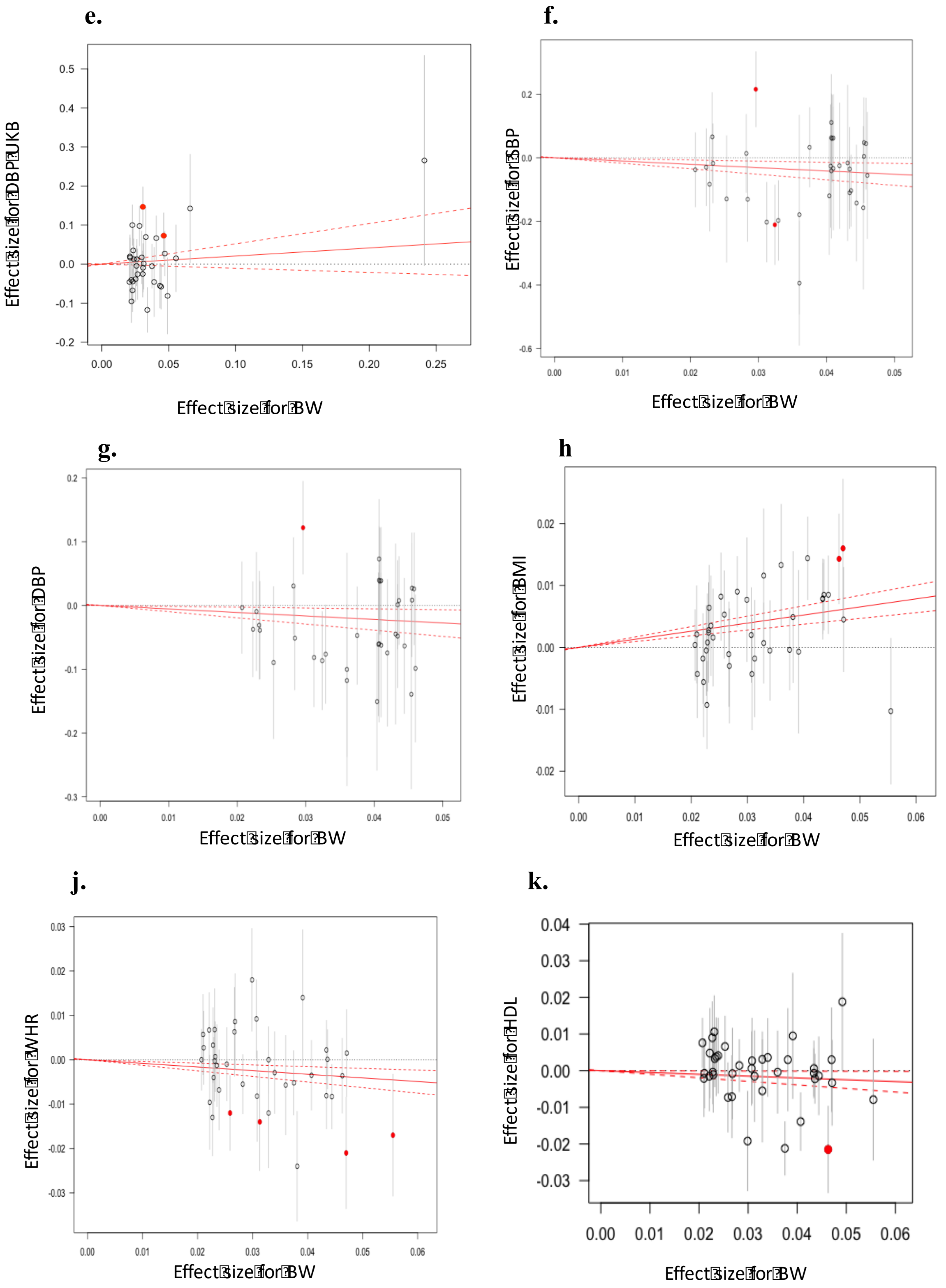
e.
Effect size for DBP UKB
Effect size for BW
f.
Effect size for SBP
Effect size for BW
g.
Effect size for DBP
Effect size for BW
h
Effect size for BMI
Effect size for BW
j.
Effect size for WHR
Effect size for BW
k.
Effect size for HDL
Effect size for BW

i.

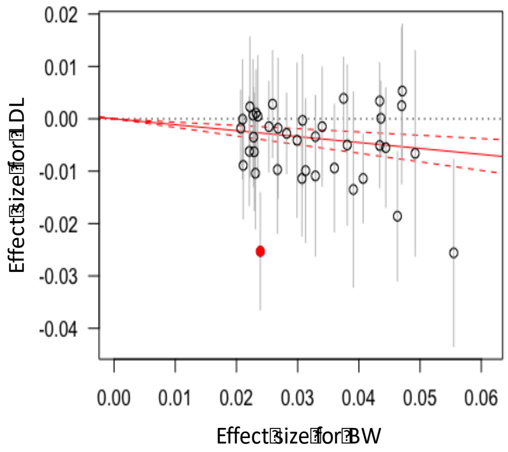
Effect size for LDL
0.02
0.01
0.00
-0.01
-0.02
-0.03
-0.04
0.00 0.01 0.02 0.03 0.04 0.05 0.06
Effect size for BW

l.

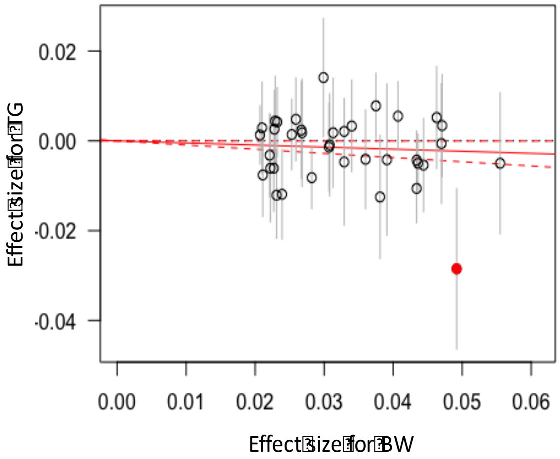
Effect size for TG
0.02
0.00
-0.02
-0.04
0.00 0.01 0.02 0.03 0.04 0.05 0.06
Effect size for BW

m.

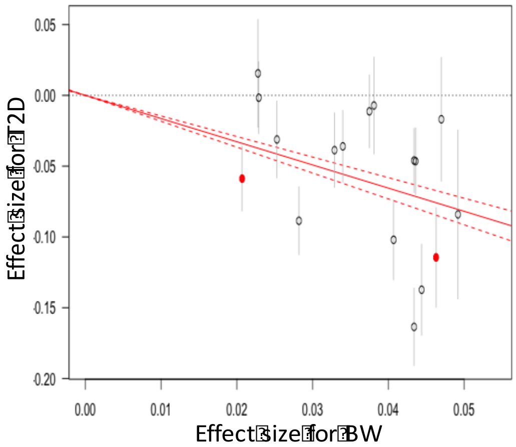
Effect size for T2D
0.05
0.00
-0.05
-0.10
-0.15
-0.20
0.00 0.01 0.02 0.03 0.04 0.05
Effect size for BW

n.

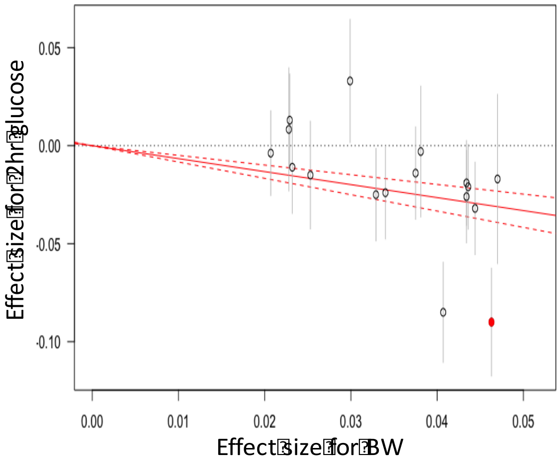
Effect size for 2hr glucose
0.05
0.00
-0.05
-0.10
0.00 0.01 0.02 0.03 0.04 0.05
Effect size for BW

o.

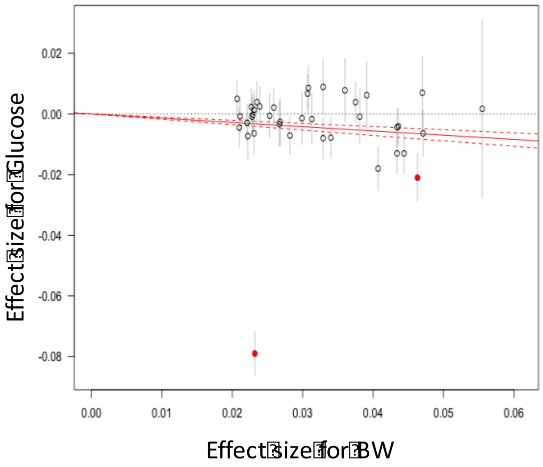
Effect size for Glucose
0.02
0.00
-0.02
-0.04
-0.06
-0.08
0.00 0.01 0.02 0.03 0.04 0.05 0.06
Effect size for BW

p.

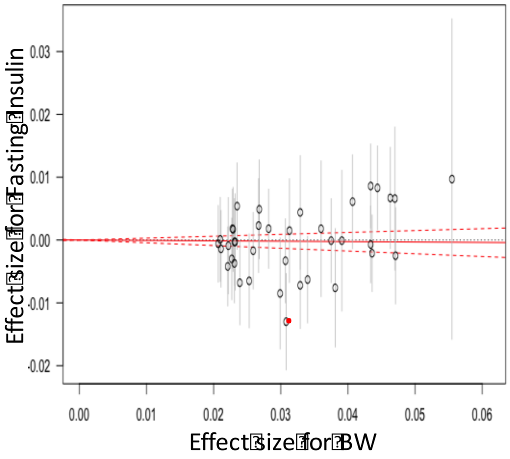
Effect size for Fasting Insulin
0.03
0.02
0.01
0.00
-0.01
-0.02
0.00 0.01 0.02 0.03 0.04 0.05 0.06
Effect size for BW

Supplemental Figure IV. Flow chart showing the different data sources used in this study.

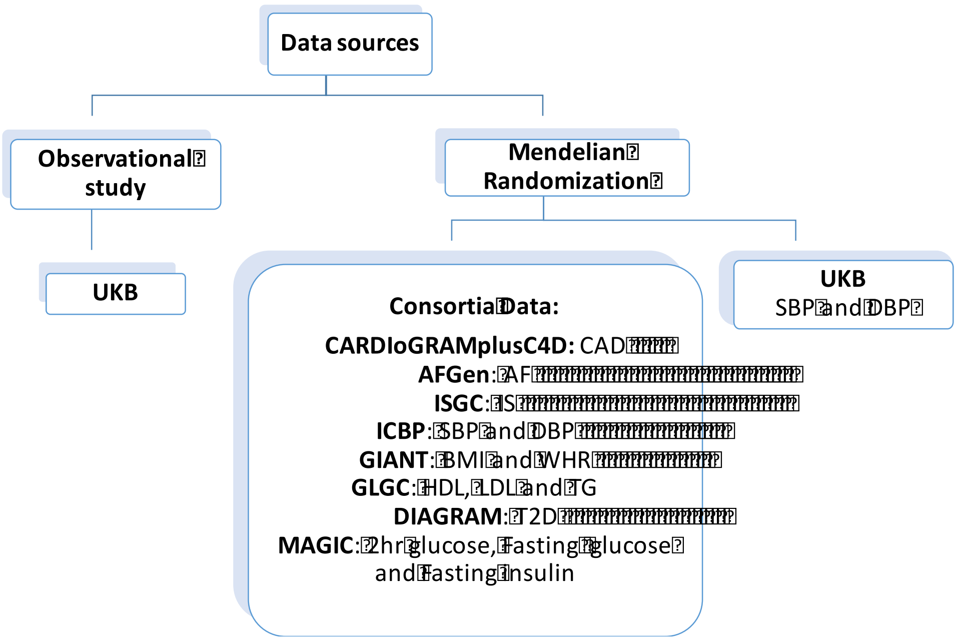

Abbreviations: UKB, UK Biobank; CAD, coronary artery disease; AF, atrial fibrillation; IS, ischemic stroke; SBP, systolic blood pressure; DBP, diastolic blood pressure; BMI, body mass index; WHR, waist-to-hip ratio; HDL, high density lipoprotein; LDL, low density lipoproteins; TG, triglycerides; T2D, type 2 diabetes; CAD, CARDIoGRAMplusC4D; AFGen, Atrial Fibrillation Genetics; ISGC, International Stroke Genetics Consortium; ICBP, International Consortium for Blood Pressure; GIANT, Genetic Investigation of ANthropometric Traits; GLGC, Global Lipids Genetic Consortium; DIAGRAM, DIAbetes Genetics Replication and Meta-analysis; MAGIC, Meta-Analysis of Glucose and Insulin related traits Consortium.

Supplemental Figure V. Relations of birth weight (BW) with **a.** coronary artery disease (CAD); **b.** atrial fibrillation (AF); **c.** ischemic stroke (IS); **d.** hemorrhagic stroke (HS); **e.** heart failure (HF). Lines are based on a regression spline of Cox proportional hazards.

a.

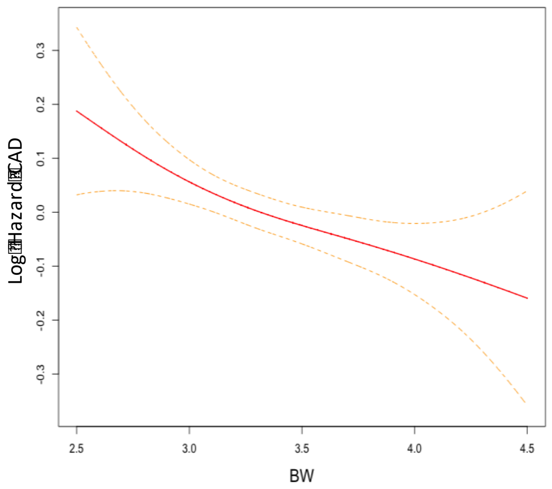

b.

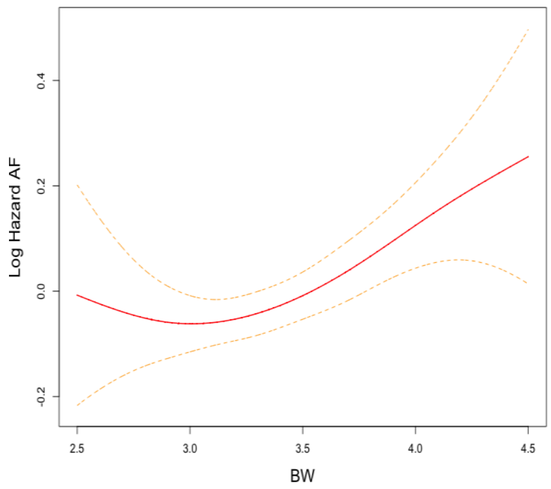

c.

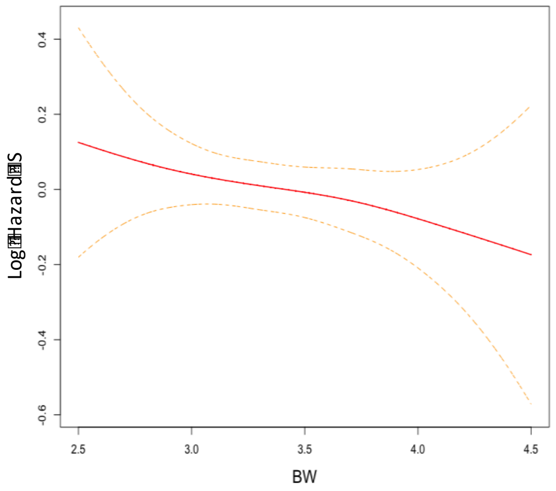

d

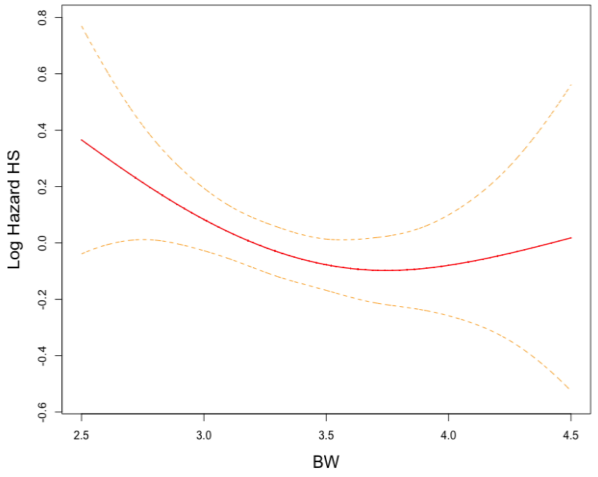

e.

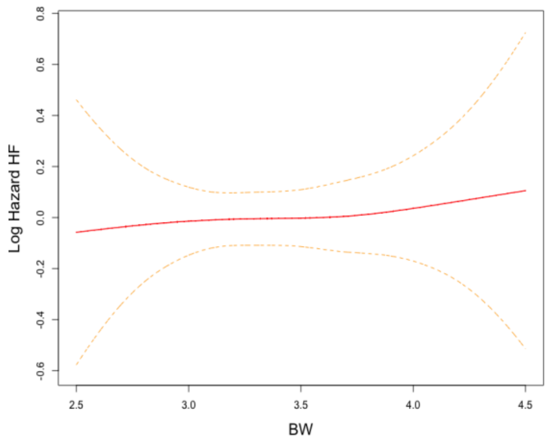

Supplemental Figure VI. Scatter plots performed across all MR methods tested in our study in addition to several penalized-robust methods (**1**) and the leave-one-out sensitivity analysis (**2**). Each black point in the forest plot represents the MR analysis (using IVW) excluding that particular SNP. The overall analysis including all SNPs is also shown for comparison. **a.** coronary artery disease (CAD); **b.** atrial fibrillation (AF); **c.** ischemic stroke (IS); **d.** systolic blood pressure in UK Biobank (SBP UKB); **e.** diastolic blood pressure in UK Biobank (DBP UKB); **f.** systolic blood pressure from International Consortium of Blood Pressure (SBP); **g.** diastolic blood pressure from International Consortium of Blood Pressure (DBP); **h.** body mass index (BMI); **i.** waist-to-hip ratio (WHR); **j.** high density lipoprotein (HDL); **k.** low density lipoproteins (LDL); **l.** triglycerides (TG); **m.** type 2 diabetes (T2D); **n.** 2-hour glucose; **o.** fasting glucose; **p.** fasting insulin.

a1.

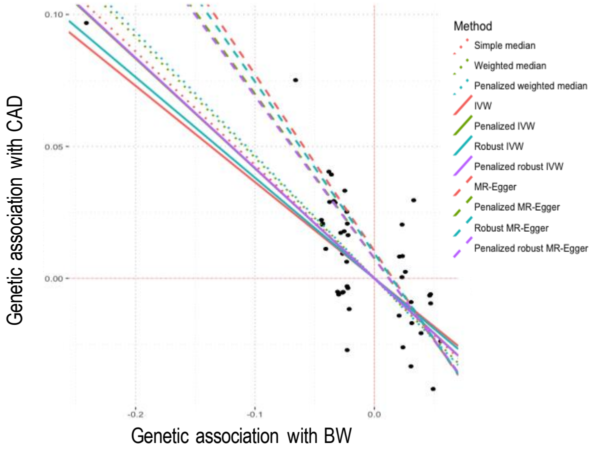

a2.

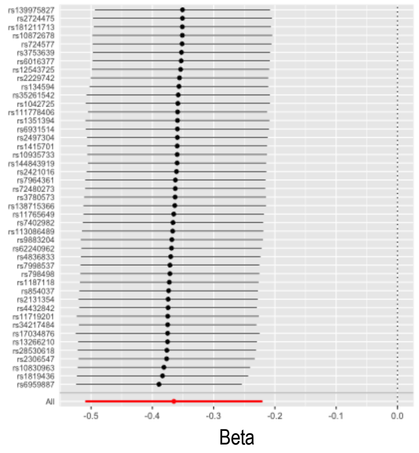

b1.

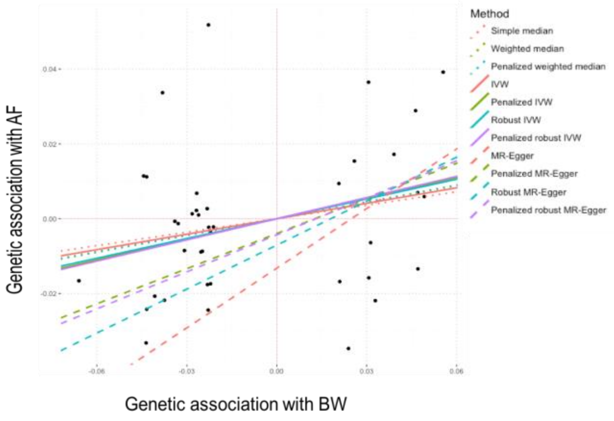

b2.

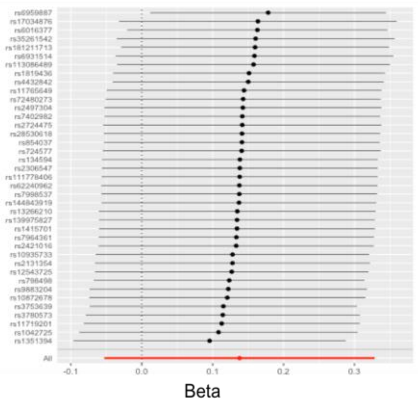

c1.

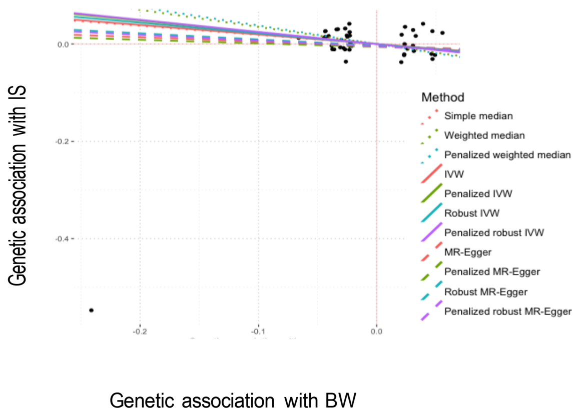

c2.

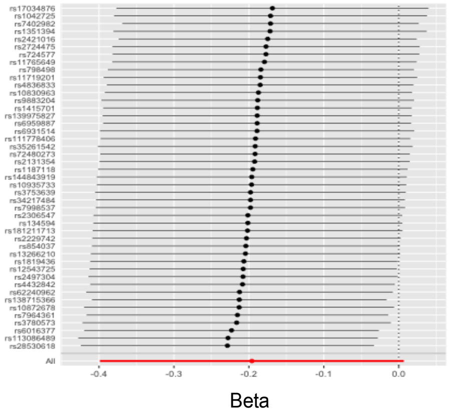

d1.

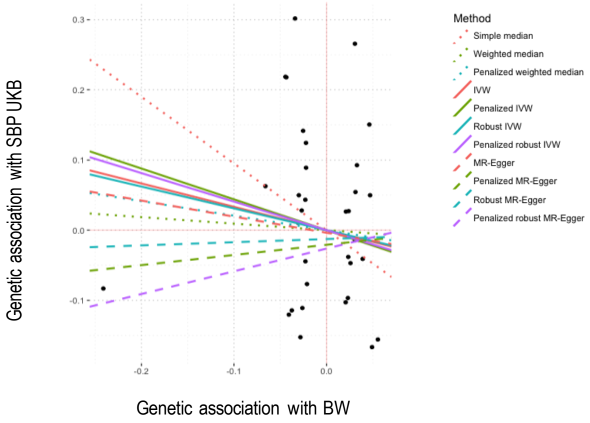

d2.

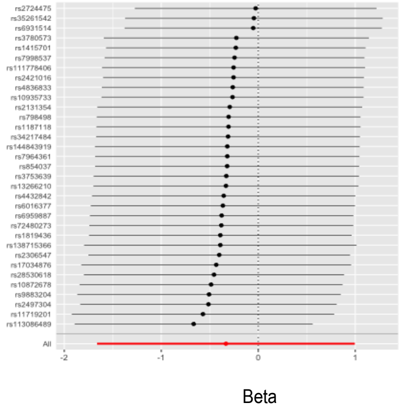

e1.

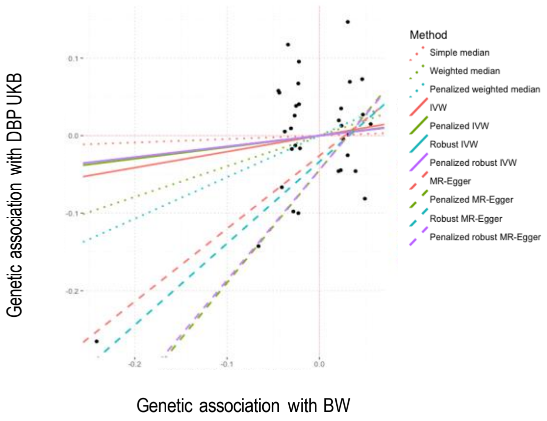

e2.

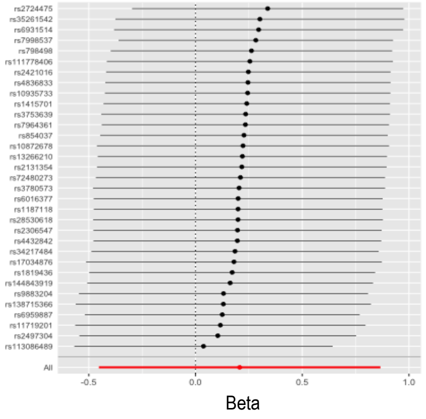

f1.

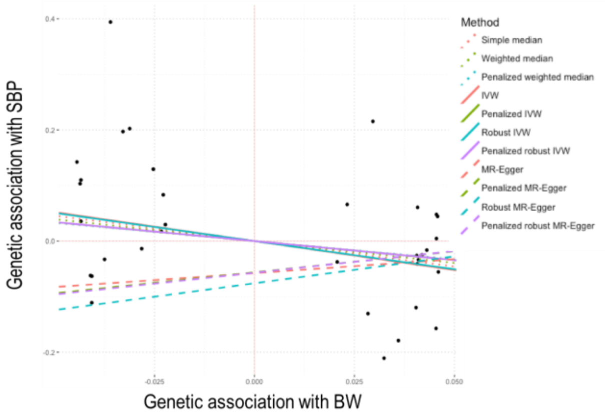

f2.

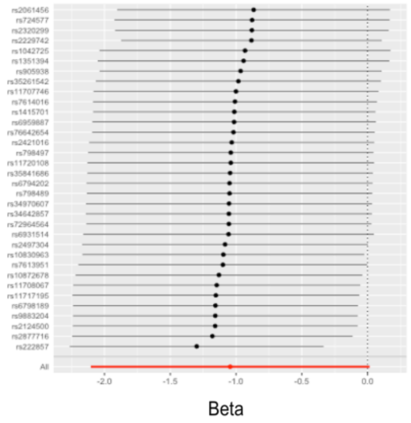

g1.

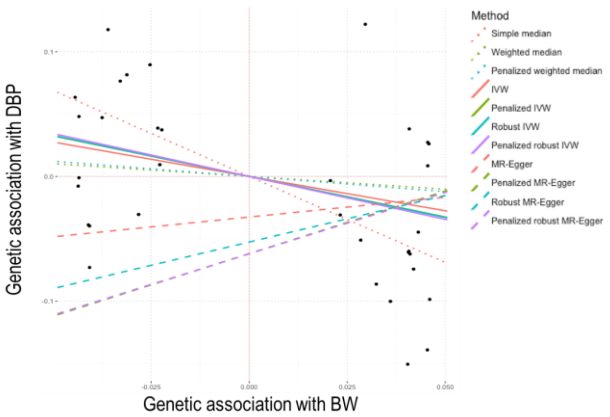

g2.

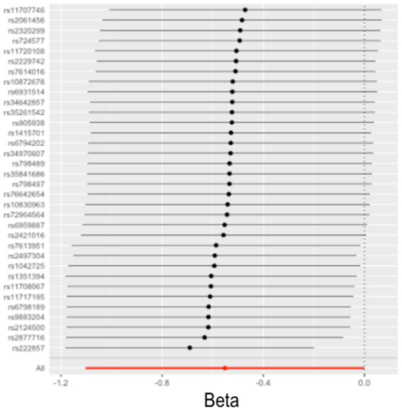

h1.

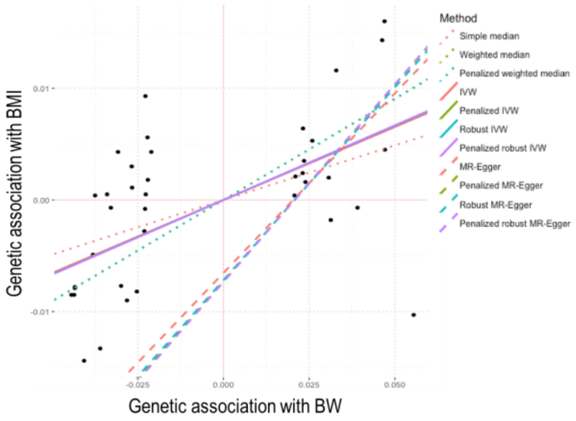

h2.

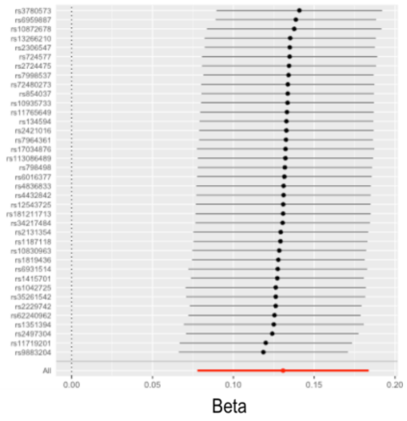

i1.

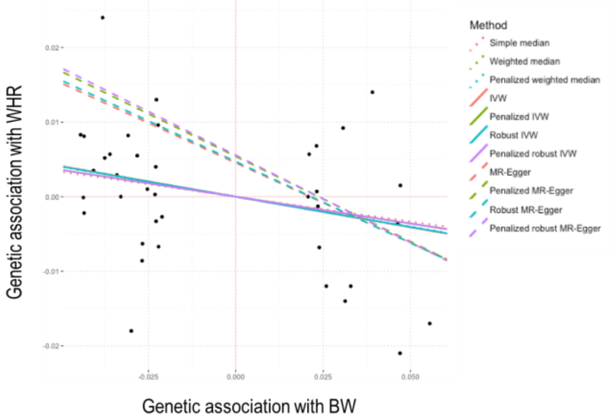

i2.

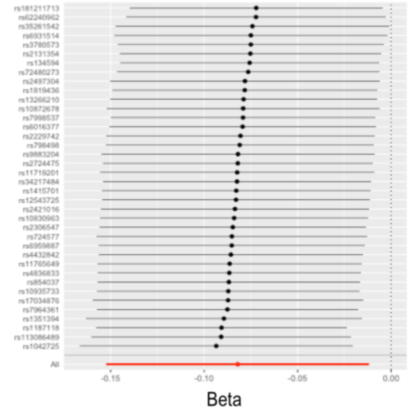

j1.

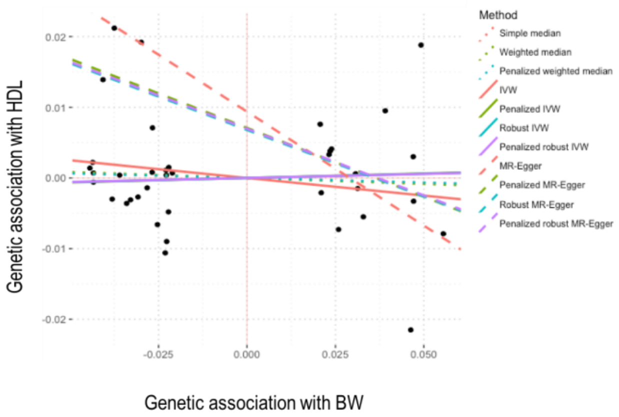

j2.

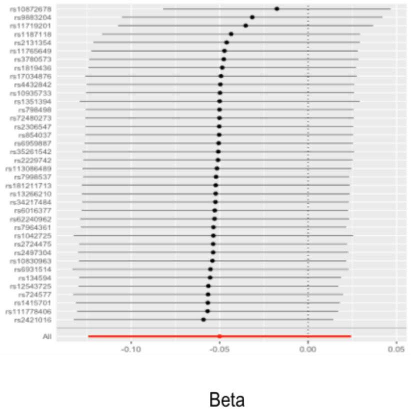

k1.

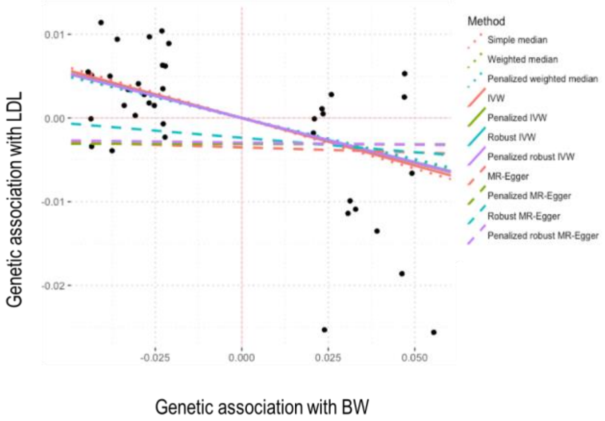

k2.

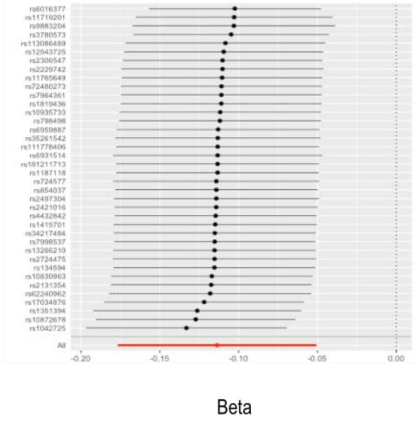

l1.

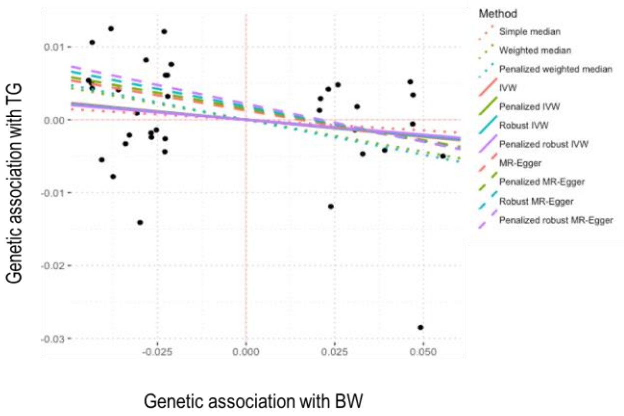

l2.

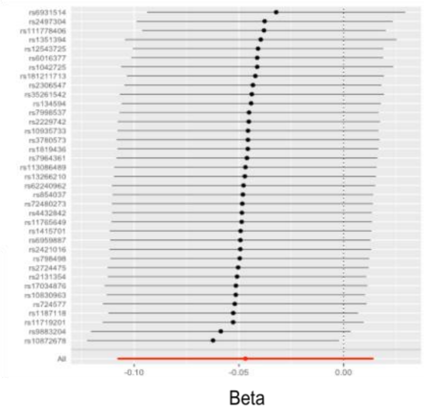

m1.

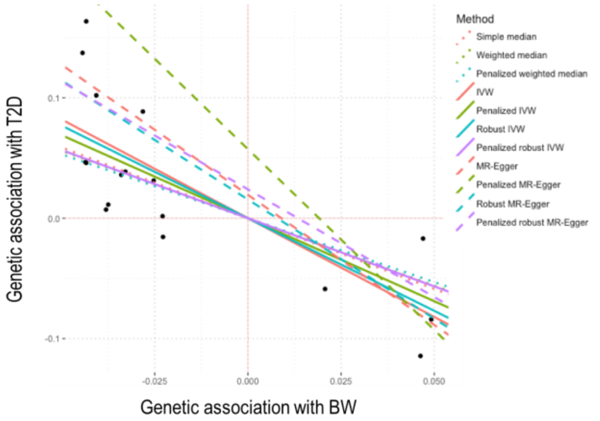

m2.

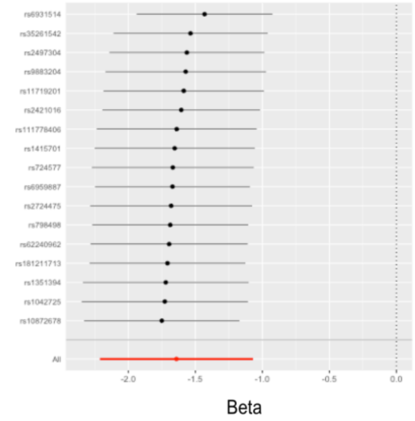

n1.

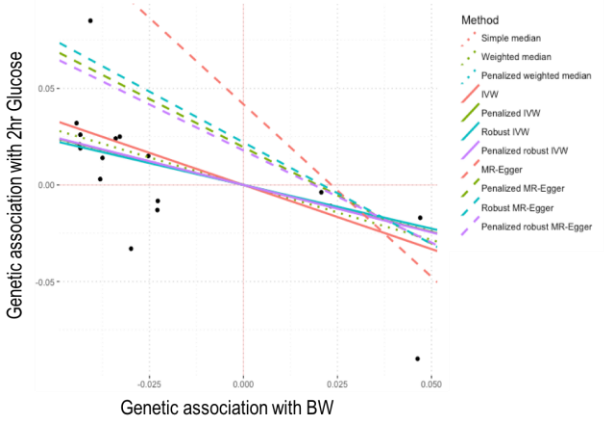

n2.

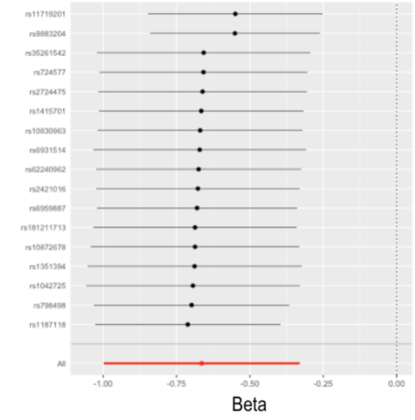

o1.

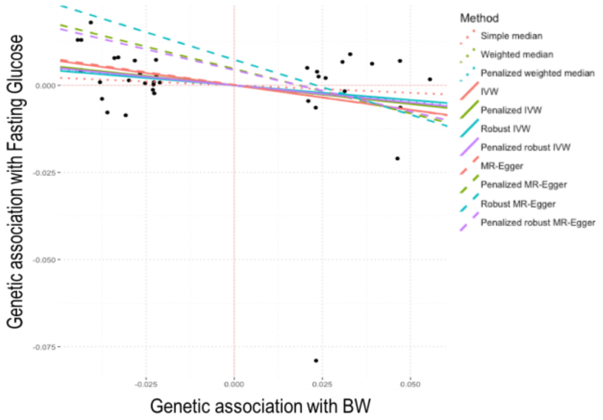

Method
Simple median
Weighted median
Penalized weighted median
IVW
Penalized IVW
Robust IVW
Penalized robust IVW
MR-Egger
Penalized MR-Egger
Robust MR-Egger
Penalized robust MR-Egger
Genetic association with Fasting Glucose
Genetic association with BW

o2.

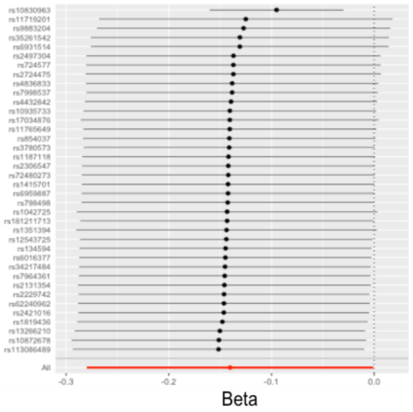

All
-0.3
-0.2
-0.1
0.0
Beta

p1.

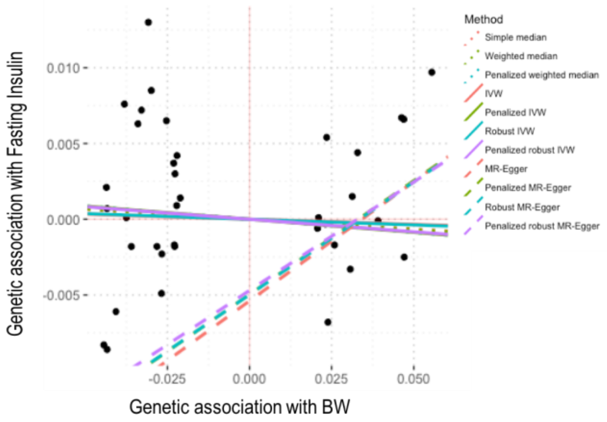

Method
Simple median
Weighted median
Penalized weighted median
IVW
Penalized IVW
Robust IVW
Penalized robust IVW
MR-Egger
Penalized MR-Egger
Robust MR-Egger
Penalized robust MR-Egger
0.010
0.005
0.000
-0.005
-0.025
0.000
0.025
0.050
Genetic association with Fasting Insulin
Genetic association with BW

p2.

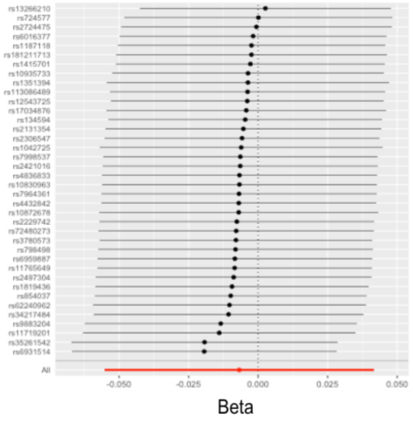

All
-0.050
-0.025
0.000
0.025
0.050
Beta

Supplemental Figure VII. Causal associations of birth weight with outcomes using Mendelian randomization analyses. **a.** Continuous outcomes: systolic blood pressure in UK Biobank (SBP UKB); body mass index (BMI), waist-to-hip ratio (WHR), high density lipoprotein (HDL); low density lipoproteins (LDL); triglycerides (TG); 2-hour glucose; fasting glucose; and fasting insulin. **b.** Binary outcomes: coronary artery disease (CAD), atrial fibrillation (AF), ischemic stroke (IS), type 2 diabetes (T2D).

a.

	Beta	95 % CI
**SBP UKB N=33**		
IVW	-0.33	(-1.66 ,1.00)
Penalized Robust IVW	-0.41	(-1.34 ,0.52)
MR-Egger	0.23	(-3.86 ,3.41)
Weighted median	-0.09	(-1.19 ,1.00)
**DBP UKB N=33**		
IVW	0.21	(-0.45 ,0.87)
Penalized Robust IVW	0.14	(-0.37 ,0.65)
MR-Egger	0.94	(-0.84 ,2.72)
Weighted median	0.40	(-0.17 ,0,96)
**BMI N=38**		
IVW	0.13	(0.08 ,0.18)
Penalized Robust IVW	0.13	(0.09 ,0.18)
MR-Egger	0.32	(0.14 ,0.50)
Weighted median	0.18	(0.12 ,0.24)
**WHR N=38**		
IVW	-0.08	(-0.15 ,-0.01)
Penalized Robust IVW	-0.07	(-0.13 ,-0.02)
MR-Egger	-0.21	(-0.47 ,0.04)
Weighted median	-0.08	(-0.15 ,-0.01)
**HDL N=38**		
IVW	-0.05	(-0.12 ,0.02)
Penalized Robust IVW	0.01	(-0.03 ,0.05)
MR-Egger	-0.32	(-0.58 ,-0.07)
Weighted median	-0.02	(-0.09 ,0.06)
**LDL N=38**		
IVW	-0.11	(-0.18 ,-0.05)
Penalized Robust IVW	-0.11	(-0.16 ,-0.05)
MR-Egger	-0.01	(-0.24 ,0.22)
Weighted median	-0.1 0	(-0.18 ,-0.02)
**TG N=37**		
IVW	-0.05	(-0.11 ,0.01)
Penalized Robust IVW	-0.04	(-0.10 ,0.02)
MR-Egger	-0.08	(-0.31 ,0.14)
Weighted median	-0.09	(-0.16 ,-0.01)
**2hr glucose N=17**		
IVW	-0.66	(-1.00 ,-0.33)
Penalized Robust IVW	-0.49	(-0.66 ,-0.32)
MR-Egger	-1.79	(-3.03 ,-0.54)
Weighted median	-0.57	(-0.82 ,-0.32)
**Fasting glucose N=38**		
IVW	-0.14	(-0.28 ,-0.01)
Penalized Robust IVW	-0.10	(-0.16 ,-0.04)
MR-Egger	-0.15	(-0.66 ,0.37)
Weighted median	-0.10	(-0.17 ,-0.03)
**Fasting insulin N=38**		
IVW	-0.01	(-0.06 ,0.04)
Penalized Robust IVW	-0.02	(-0.06 ,0.03)
MR-Egger	0.16	(-0.01 ,0.33)
Weighted median	-0.01	(-0.07 ,0.05)

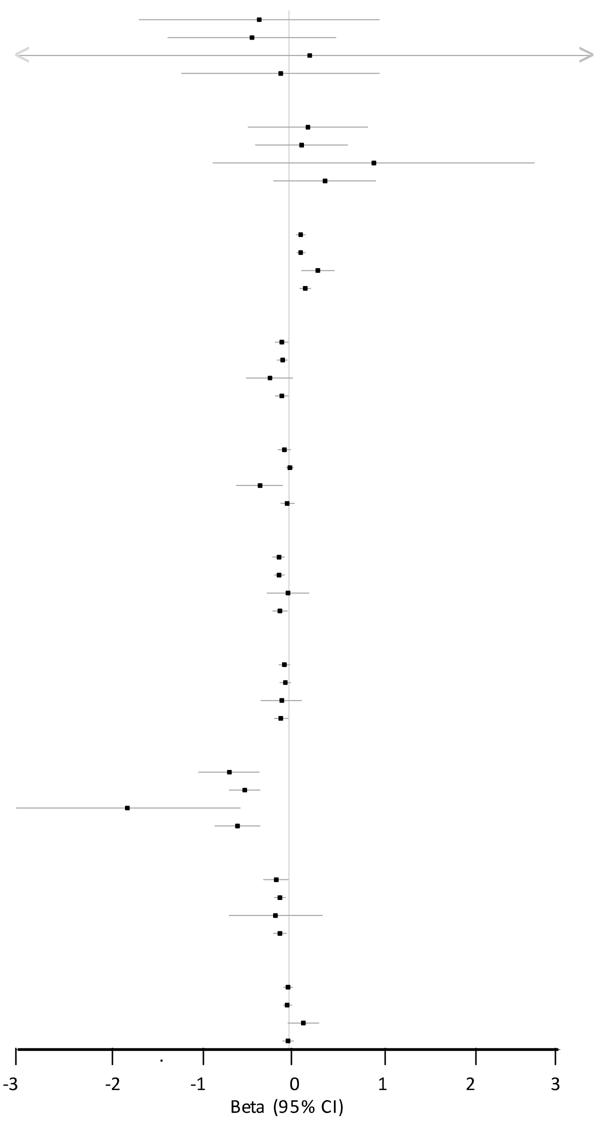

b.

CAD N=45	OR	95% CI
IVW	0.69	(0.60, 0.80)
Penalized Robust IVW	0.66	(0.59, 0.74)
MR-Egger	0.51	(0.33, 0.79)
Weighted median	0.63	(0.54, 0.74)
**AF N=39**		
IVW	1.15	(0.95, 1.39)
Penalized Robust IVW	1.20	(1.04, 1.40)
MR-Egger	1.70	(0.87, 3.31)
Weighted median	1.16	(0.92, 1.45)
**IS N=45**		
IVW	0.82	(0.67, 1.01)
Penalized Robust IVW	0.78	(0.65, 0.93)
MR-Egger	0.92	(0.46, 1.84)
Weighted median	0.70	(0.54, 0.91)
**T2D N=17**		
IVW	0.19	(0.11, 0.34)
Penalized Robust IVW	0.25	(0.19, 0.34)
MR-Egger	0.12	(0.01, 1.27)
Weighted median	0.33	(0.22, 0.48)

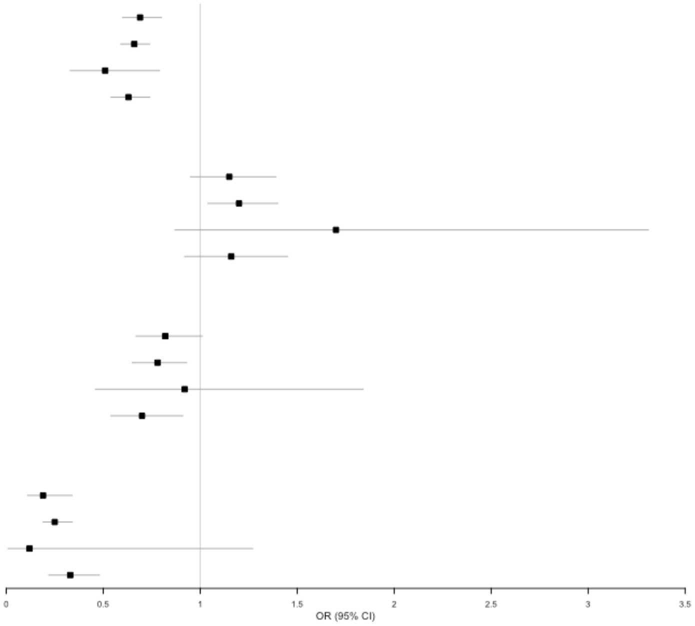

Mendelian randomization analyses based on the 46 variants included in the instrument variable 2 using data sources listed in Table 2. All effects (beta or OR) are given in original units as provided by the consortia.

Abbreviations: DBP, diastolic blood pressure; SBP, systolic blood pressure; UKB, UK Biobank; BMI, body mass index; WHR, waist-to-hip ratio; HDL, high density lipoprotein; LDL, low density lipoproteins; TG, triglycerides; CAD, coronary artery disease; AF, atrial fibrillation; IS, ischemic stroke; T2D, type 2 diabetes; IVW, Inverse variance weighting method; N, number of SNPs; OR, odds ratio; CI, confidence interval.
